# Overexpression of *MEOX2* and *TWIST1* Is Associated with H3K27me3 Levels and Determines Lung Cancer Chemoresistance and Prognosis

**DOI:** 10.1371/journal.pone.0114104

**Published:** 2014-12-02

**Authors:** Federico Ávila-Moreno, Leonel Armas-López, Aldo M. Álvarez-Moran, Zoila López-Bujanda, Blanca Ortiz-Quintero, Alfredo Hidalgo-Miranda, Francisco Urrea-Ramírez, R. María Rivera-Rosales, Eugenia Vázquez-Manríquez, Erika Peña-Mirabal, José Morales-Gómez, Juan C. Vázquez-Minero, José L. Téllez-Becerra, Roberto Ramírez-Mendoza, Alejandro Ávalos-Bracho, Enrique Guzmán de Alba, Karla Vázquez-Santillán, Vilma Maldonado-Lagunas, Patricio Santillán-Doherty, Patricia Piña-Sánchez, Joaquin Zúñiga-Ramos

**Affiliations:** 1 Universidad Nacional Autónoma de México (UNAM), Facultad de Estudios Superiores (FES)-Iztacala, Biomedicine Research Unit (UBIMED), Cancer Epigenomics Laboratory 12, Tlalnepantla, Mexico State, Mexico; 2 Instituto Nacional de Enfermedades Respiratorias (INER), Mexico City, Mexico; 3 Johns Hopkins University, Medical Institutions, Maryland, Baltimore, United States of America; 4 Instituto Nacional de Medicina Genómica (INMEGEN), Mexico City, Mexico; 5 Unidad de Investigación Médica en Enfermedades Oncológicas (UIMEO), Instituto Mexicano del Seguro Social (IMSS), Centro Médico Nacional (CMN), Siglo XXI, México City, México; Roswell Park Cancer Institute, United States of America

## Abstract

Lung cancer is the leading cause of death from malignant diseases worldwide, with the non-small cell (NSCLC) subtype accounting for the majority of cases. NSCLC is characterized by frequent genomic imbalances and copy number variations (CNVs), but the epigenetic aberrations that are associated with clinical prognosis and therapeutic failure remain not completely identify. In the present study, a total of 55 lung cancer patients were included and we conducted genomic and genetic expression analyses, immunohistochemical protein detection, DNA methylation and chromatin immunoprecipitation assays to obtain genetic and epigenetic profiles associated to prognosis and chemoresponse of NSCLC patients. Finally, siRNA transfection-mediated genetic silencing and cisplatinum cellular cytotoxicity assays in NSCLC cell lines A-427 and INER-37 were assessed to describe chemoresistance mechanisms involved. Our results identified high frequencies of CNVs (66–51% of cases) in the 7p22.3–p21.1 and 7p15.3–p15.2 cytogenetic regions. However, overexpression of genes, such as *MEOX2*, *HDAC9*, *TWIST1* and *AhR*, at 7p21.2–p21.1 locus occurred despite the absence of CNVs and little changes in DNA methylation. In contrast, the promoter sequences of *MEOX2* and *TWIST1* displayed significantly lower/decrease in the repressive histone mark H3K27me3 and increased in the active histone mark H3K4me3 levels. Finally these results correlate with poor survival in NSCLC patients and cellular chemoresistance to oncologic drugs in NSCLC cell lines in a *MEOX2* and *TWIST1* overexpression dependent-manner. In conclusion, we report for the first time that *MEOX2* participates in chemoresistance irrespective of high CNV, but it is significantly dependent upon H3K27me3 enrichment probably associated with aggressiveness and chemotherapy failure in NSCLC patients, however additional clinical studies must be performed to confirm our findings as new probable clinical markers in NSCLC patients.

## Introduction

Lung cancer remains the leading cause of cancer-related death in both the USA [1] and worldwide, with the non-small cell (NSCLC) subtype accounting for the majority of cases in smokers and non-tobacco-related patients [2]. Late diagnoses and inefficient therapeutics contribute to poor prognoses and to average 5-year survival rates less than 15% [1], [3], [4], [5].

NSCLC is characterized by genomic abnormalities, which include common somatic DNA copy number variations (CNVs). Comparative genomic hybridization (CGH) by spectral karyotyping [6] or low-resolution genomic DNA microarray assays [7], [8], [9] have highlighted losses/deletions at 2q36–37, 3p21, 8p22, 9p21–22, 9q22, 13q22 and 17p12–13, and gains/amplifications at 1q23, 1q31, 3q25–27, 5p13–14, 6p, 7q and 8q23–24; however, few of these alterations have been shown to be involved in tumor aggressiveness [9] or the clinical progression of NSCLC.

Recently, international consortiums for the study of lung cancer have used high-density SNP arrays (250 K) to describe CNVs, such as 5p15.33, 7p11.2, 14q13.3 and 17q12, as well as microdeletions at 5q11.2, 7q11.22 and 9p23 [10]. Some of these CNVs, such as gains at 14q13.3 containing the *NKX2-8* [11] and *NKX2-1* genes [10], have been shown to be functionally involved in growth, survival and tumorigenic activity in NSCLC [10], [11]. Increases in the CNV at the 5p13.2 locus have been proposed as a new molecular marker of early pre-invasiveness [12]. In addition, a genetic signature of mRNA overexpression from 7q11.23, 14q23.2 and 17q21.2 (*STX1A*, *HIF1A* and *CCR7*, respectively) has been identified as a predictive progression-prognosis signature during the early clinical stages of NSCLC patients [13].

Poor prognoses have been associated with the 7p locus, which may be due, in part, to increased CNV and the overexpression of potential oncogenes at 7p15.3–11.2, which have been described in 56% of NSCLC cell lines [14]. Also, gains at 7p12–21 have been identified in nearly 50% of NSCLC patients with metastatic disease [15], which may represent one of the karyotypic evolution models of NSCLC based on gains of chromosome 7 [16]. Moreover, epigenetic aberrations, such as DNA hypermethylation at 7p15.2 [17], may represent relevant regulatory mechanisms and/or markers, not only for lung cancer biology [16] but also for NSCLC progression, due to their presence during the early [17] and late clinical stages [10], [11], [15]. However, to date, the molecular relationships between high-frequency CNVs, epigenetic aberrations, patient prognoses and oncologic failure in NSCLC have not yet been fully investigated.

In the present study, we attempted to correlate potential epigenetic modifications with the pattern of DNA copy number changes in NSCLC through the use of high-resolution tiling SNP arrays (500 K). We identified amplifications at the 7p22.3–p22.1, 7p21.3–p21.1 and 7p15.3–p15.2 cytogenetic regions in range of 66–51% of our cases. These amplifications resulted in *MEOX2*, *HDAC9*, *TWIST1* and *AhR* overexpression despite the little increased levels of DNA methylation. However, the significantly lower enrichment of the repressive histone marker H3K27me3 and the significantly increased activation of H3K4me3 at both the *MEOX2* and *TWIST1* promoter regions were correlated with poor clinical prognoses. Therefore, as we demonstrated in NSCLC cell line models, cisplatinum chemoresistance correlates with an over-expression of *MEOX2* and *TWIST1* genes, mainly due to the loss of the histone repressive marks H3K27me3. Our results suggest that epigenetic mechanisms exceed genetic (CNV) aberrations at 7p21 and complement them, thereby contributing to aggressiveness and the failure of oncologic therapy in NSCLC patients.

## Materials and Methods

### Sample Selection

A total of 55 lung tumors (LT), 15 adjacent non-involved lung matched tissues (LNAT) and 20 lung precursor lesions (LP) from Fresh Frozen (FF) and Formalin-Fixed and Paraffin Embedded (FFPE) tissues processing methods, were collected from the Thoracic Surgical Service and Pathology Service at the National Institute of Respiratory Diseases (INER). Additionally, the NSCLC cell lines SK-MES-1, A-549, A-427, were obtained from the American Type Culture Collection (ATCC, Manassas, VA, USA) and INER-37, INER-51 were obtained from Mexican Mestizo patients, as previously reported [18], [19].

### Ethics Statement

The Institutional Review Board (IRB) of the National Institute of Respiratory Diseases (INER) approved the protocols with register B17-07 and B09-08, and from the Universidad Nacional Autónoma de México (UNAM), FES-Iztacala, Biomedicine Research Unit reviewed and approved the protocols for these genetic and molecular biology studies. Informed consent was obtained from patients with diagnosis of LT and undergoing surgical procedures. All subjects signed the informed consent letter for these research studies at thoracic surgery service, INER, and they authorized the storage of their biological samples at INER and UNAM repositories for this and future studies. In this study we did not collected samples from minors/children, only young adults older than 18 years were included.

### SNP Mapping Array 500 K Hybridization and Bioinformatics Analyses

A total of 30 LNAT, LT, LP and NSCLC cell line DNA samples were included in the array hybridization platform (SNP 500 K) and in some cases for SNP 6.0 arrays, according to the manufacturer's recommended instructions (Affymetrix, Santa Clara, CA, USA), and analyzed using the previously described method [20] by comparing 300 human healthy donors from the Mexican Mestizo population Haplotype Map database previously described [21]. Affymetrix SNP arrays data (Affymetrix Console Version 4.1.3), were imported, normalized and increases of CNV were identified using Partek Genomics Suite Program Defaults and Partek Genome View Tools (Partek, Saint Louis, MI, USA), when the average of 20 SNP exceeded a copy number ratio of 2.5 or was less than 1.5 (*FDR*≤0.02). Pathway prediction analyses from the genes with copy number changes in our NSCLC samples (File S1) were performed using the Software Ingenuity System Program (Version 7.0). The list of aberrant genes included in our cellular pathway prediction analyses, were selected based on the availability of expression profiles in human lung tissues and lung carcinoma tissues, using data in EntrezGene browsing (http://www.ncbi.nlm.nih.gov/gene) and web browser GeneCards Human Gene Database (http://www.genecards.org/). SNP array data included in the present study were submitted and approved by the Gene Expression Omnibus (GEO) database with the GEO official number: GSE62407.

### Immunohistochemical Assays in Histological Samples

Immunohistochemical analyses were performed on LNAT, LT and LP tissue samples fixed with formaldehyde 10% or Hope I/II reactive Method (Innovative Diagnostik-System, Germany), and subsequently using 1–2 micrograms of specific antibodies anti-MEOX2, anti-TWIST1, anti-HDAC9, and anti-EVX1 (Santa Cruz Biotechnology, Dallas, Texas, USA), by incubation in a humidity chamber and in addition of the antigen-specific reaction with avidin-biotin peroxidase/diamino-benzidine system (Dako, Carpinteria, CA, USA). A Ventana System device (Roche, Tucson, Arizona, USA) and a transmitted light microscopy study (Leica, Bannockburn, IL, USA), were used to obtain photomicrographs at 40X and 80X magnification.

### Real-Time PCR for mRNA Expression and DNA Quantification Assays

For the mRNA expression analyses, cDNA was synthesized from 2 µg of total RNA using the High Capacity Kit (Life Technologies Corporation, Applied Biosystems, Foster City, CA, USA). cDNA and genomic DNA were also amplified using SYBR Green qPCR assays and oligonucleotide sets designed by Primer Design and/or Vector NT software programs, and synthesized by Sigma-Genosys (Sigma-Aldrich, St. Louis, MO, USA), described for mRNA expression assays in Table S1, and for DNA promoter sequences analysis in Table S2.

Quantitative real-time PCR (qPCR) was performed on a Step One Plus and 7500 Device (Life Technologies Corporation, Applied Biosystems, Foster City, CA, USA), and in a LightCycler 480 System (Roche, Mannheim, Germany), using Power SYBR Green (Life Technologies Corporation, Applied Biosystems, Foster City, CA, USA) and Maxima SYBR Green qPCR Master Mix (Thermo Fisher Scientific, Fermentas, Life Science, Foster City, CA,).

Conditions for relative quantitative PCR reaction using Fermentas SYBR Green were as follows, one cycle of 50°C for 2 min, one cycle of 95°C for 10 min, 40 cycles of 95°C for 15 s, 60°C for 30 s, and additional extension step at 60°C for 30 s. While for Applied Biosystems Power SYBR Green were as follows: one cycle of 50°C for 2 min, one cycle of 95°C for 10 min, 40 cycles of 95°C for 15 s, 60°C for 30 s, and 72°C for 30 s. At the end of the PCR reaction, samples were subjected to a melting analysis to confirm specificity of the amplicon, and β-actin gene was used as housekeeping for normalization analysis.

### Restriction Enzyme Methylation-Sensitive Assays

Methylation status of genomic DNA (200 ng) that was derived from LNAT, LT and LP tissues and the lung cancer cell lines was examined using 0.5 units each one of a combination of methylation-sensitive restriction enzymes *HpaII*, *HpyCH4IV* and/or *AciI* (New England Biolabs, Ipswich, MA, USA) and incubating at 37°C, for 4 h. Followed by methylation-sensitive PCR amplification assays using oligonucleotide sets (Table S2), designed inside of the predicted CpG islands of the promoter's target genes (*MEOX2*, *HDAC9, TWIST1*, and *AhR*), following criteria as island size >100, and GC Percent >50.0, using MethPrimer to enclose as many restriction sites to attain the enzymes sensitive to methylation status, inside the amplicon and indicated at Table S3.

### Methylation Sensitive PCR Assays

Methylation status (%) was calculated based on Methylation Sensitive PCR assays, by using oligonucleotide sets containing CpG sites inside on the amplicon at promoter's target genes studied (*MEOX2*, *HDAC9, TWIST1*, and *AhR*) described at Table S3. Briefly, 50 ng of digested DNA were used as input on 25 µl of total PCR reaction, and product sizes analyzed, according to the Table S3, follows the next PCR conditions: one cycle of 95°C for 10 min, 40 cycles of 95°C for 15 s, 55°C for 30 s and 72°C 45 s.

Furthermore, human lung (NSCLC) cell lines A-427 and INER-37 were used to standardize the enzymatic restriction conditions, developing *in vitro* methylation assays using CpG methyl-transferase (M.SssI) and S-adenosyl methionine (SAM), by incubating at 37°C for 4 h. Standard methylated DNA and human sperm DNA extracted from healthy donors were used as hyper-methylated and hypo-methylated DNA controls for enzymatic restriction assays.

### *In Vitro* DNA Methylation and Histone Modification Inhibition Assays

The A-427 and INER-37 human NSCLC (adenocarcinoma) cell lines (5×10^5^ cells) were treated *in vitro* with 5′-aza-deoxycytidine [4 µM], to inhibit *de novo* DNA methylation at sequence promoters, and/or with TSA [300 nM], to inhibit the activity of histone deacetylases (HDACs) during 48 h.

### Chromatin Immunoprecipitation (ChIP)

Fresh frozen LT (3 mm^3^) were pulverized under liquid nitrogen freezing and fixed with 1% formaldehyde during 10 min, and immediately neutralized with 0.125 M glycine during 5 min. Resulting cells were washed with 1% PBS, and lysed with the lysis buffer (50 mM HEPES-KOH pH 7.5, 140 mM NaCl, 1 mM EDTA pH 8, Triton X-100 1%, 0.1% Sodium Deoxycholate, 0.1% SDS) containing protease inhibitor *cOmplete Mini* 1 tablet in 10 ml (Roche, Indianapolis, IN, USA).

Chromatin from LT samples was sonicated using 10 pulses of 20 seconds w/u 60 watts. 10 µg of chromatin was immunoprecipitated (ChIP) using the commercial kit EZ-Magna ChIP G (Millipore, Temecula, CA, USA), and using 2.5 µg of anti-H3K27Ac, anti-H3K4me3, anti-H3K27me3, anti-H3K9me3 and anti-RNA Pol II activated antibodies (AbCam, Cambridge Science Park, Cambridge, UK). 1 µg of anti-mouse IgG was used as a negative control for ChIP (Millipore, Temecula, CA, USA). Integrity and quality of promoter sequences of Immuno-precipitated DNA (IP-DNA) were assessed by PCR amplification product of 166 bp for the promoter sequence of the gene GADPH as recommended by the supplier (Millipore, Temecula, CA, USA).

### IP-DNA Amplification and ChIP Quantitative Analyses Using qPCR Assays

Immunoprecipitated DNA (IP-DNA) 20 ng was linearly amplified using the Whole Genome Amplification (WGA1) Kit (Sigma, St. Louis, MO, USA), and after that was purified by MiniElute columns (QIAGEN, Hilden Renania-Westfalia, Germany).

The IP-DNA obtained from both LT samples and LT cell lines were analyzed by qPCR absolute quantification using LightCycler 480 System (Roche, Mannheim, Germany), and SYBR Green Master Mix (Thermo Fisher Scientific, Fermentas Life Science, Foster City, CA, USA) and oligonucleotide sets for the targets and controls (Table S2).

IP-DNA 20 ng were used per reaction, likewise amplification of standard curves were developed, through serial dilutions of native DNA from peripheral blood mononuclear cells of healthy donors (100 ng, 10 ng, 1 ng, 0.1 ng, and 0.01 ng), and using oligonucleotide sequences targets (Table S2), and oligonucleotide sequence controls as C-fos promoter taken from Chromatin Immunoprecipitation kit Magnify System (Invitrogen, Life Technologies, Carlsbad, CA, USA).

### Cellular Cytotoxicity Assays

Cellular viability analyses were performed in 96-well plates using 3-(4,5-dimethylthiazol-2-yl)-5-(3-carboxymethoxyphenyl)-2-(4-sulphophenyl)-2H-tetrazolium (MTS) (Promega, Madison, WI, USA) in the absence or presence of the oncologic drug cisplatinum. To do this, 96-well plates were seeded with 3,000 to 5,000 cells in 200 µl of RPMI-1640 medium for 48 h and, subsequently, incubated with 10 µl of MTS [5 mg/ml] for 3 h, for later reading at 550 nm and/or 570 nm, using the formula: cell viability  =  (O.D. of the sample/Control O.D.) ×100. Likewise, drug resistance curves were developed in a dose-response dependent manner using serial dilutions of the cancer drug cisplatinum.

### Genetic Silencing (siRNA Transfection Assays)

We used small interfering RNAs (siRNAs) directed against *MEOX2* (sc-106233), *TWIST1* (sc-38604) and non-human related mRNA as a negative control (sc-37007 and sc-44230) all obtained from Santa Cruz Biotechnology (Dallas, Texas, USA). Briefly, cell cultures with 3% of antibiotic-free FCS were incubated in 96-well plates during 12 hr and then transfected with RNAi Reagent, Lipofectamine (Life Technologies Corporation, Invitrogen, Foster City, CA, USA) and siRNAs at 50 nM, in a total volume of 100 µl.

### Western Blot

Proteins were extracted and purified by the method of TRIZOL (Invitrogen), suspended in SDS 5% with protease inhibitors (complete mini, Roche, Indianapolis, IN, USA), and quantified by the Lowry method. Total proteins (30 µg) were subjected to vertical electrophoresis in acrylamide gels 12%, and then were transferred to nitrocellulose membranes using Trans-Blot-Turbo equipment/Transfer System (BioRad, Hercules, CA, USA). Membranes were incubated overnight at 4°C, blocking with low fat milk 5% in PBS 1X/Tween20 0.1%, and incubated 2 hr with antibodies (MEOX2) 1∶1500, (TWIST1) 1∶1500, (β-actin) 1∶3000, or (GAPDH) 1∶3000 (Santa Cruz Biotechnology, Dallas, Texas, USA). Membranes were incubated during 1 hr with a secondary antibody (anti mouse HRP/anti rabbit HRP) 1∶10000, at room temperature and revealed with Luminol 1 min at room temperature using C-Digit scanner (Li-cor, Lincon, Nebraska, USA) and Hypercassette and Films (Amersham, Chalfont, Buckinghamshire, England).

### Statistical Analyses

Fisher's exact test, *t*-test and Mann Whitney-U statistical tests were used to analyze the differences between groups of patients, *p* values ≤0.05 were considered statistically significant. We also used Log-Rank (Mantel Cox) and Gehan-Breslow-Wilcoxon survival curve tests. All statistical analyses were performed using SPSS statistical software for Mac-OS X (version 11.0) and GraphPad (version 5.0).

## Results

### Chromosomal Microaberrations and Increased Copy Number Variations (CNVs) with High Frequencies at 7p22–21 and 7p15 in Lung Cancer Patients

**Figure 1:**
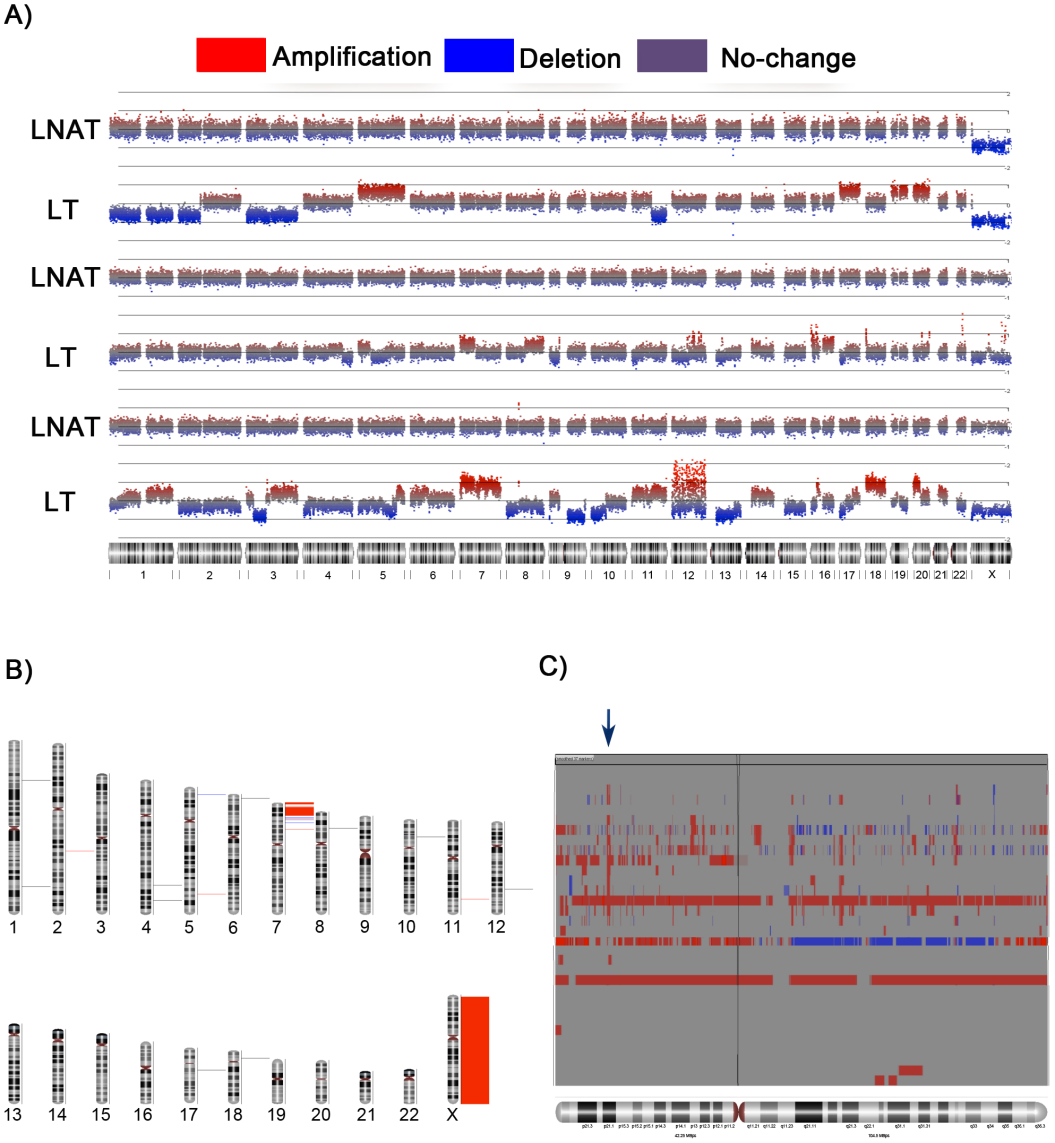
Genome-wide analysis of normal lung and paired tumors. Focal analysis of the whole genome and chromosome 7. (A) A whole-genome amplification and a bioinformatics segmental analysis of three lung cancer patients was conducted using matched, histologically adjacent non-involved lung tissue (LNAT) and lung carcinomas (LT). (B) Whole-genome and focal analyses of normal lung, lung precursor lesions and lung carcinomas (18–14 of 27 lung lesions with high CNV). (C) A focal analysis of chromosome 7 revealed a high frequency of CNV at region 7p21 (arrow) in precursor lung lesions and lung carcinomas.

In order to identify the most frequent genetic microaberrations with its probable genetic expression profiles and epigenetic aberrations in lung cancer patients, LNAT-, LP-, LT- and NSCLC cell line-derived DNA samples (n = 30) were subjected to genomic hybridization assays on SNP 500 K microarrays. The bioinformatics segmental analysis allowed the identification of chromosomal aberrations and increased CNVs in the LT-derived samples, which were not observed in the LNAT-derived matched tissues. Typical chromosomal aberrations in lung cancer including 3p deletions; amplification of multiple regions on chromosomes 5, 12, 14, 16, 18, 19 and 20; and amplification/deletion of several regions of chromosome 8 were identified (Figure 1A).

A high frequency of CNV using focal genomic analyses at 7p22.3–p22.1, 7p21.3–p21.1 and 7p15.3–p15.2 (Figure 1B) were detected. In particular, we observed DNA amplifications at the 7p21.1 locus in the LT- and LP-derived samples in nearly 55% of cases (15/27 lung lesions), including 3 of 4 precursor lesions (Figure 1C and Figure S1), which were not observed in the LNAT-derived samples (Figure 1C).

Furthermore, we confirmed increases in CNV at the 7p locus in both the LP- and LT-derived samples using the SNP 6.0 platform (Figure S2). The 39 genes with the highest rates of change in CNV are shown in Table S4. These changes were located in the 7p22.3–p22.1 and 7p21.3–p21.1 cytogenetic regions, and they included *ZNF12*, *RPA3*, *MEOX2*, *BZW2*, *TSPAN13*, *AGR2*, *AGR3*, *AhR*, *TWIST1* and *HDAC9*. Changes in *TWISTN*, *HNRNPA2*, *CBX3*, the *HOXA* cluster and *EVX1*, which are located at 7p15.3–p15.2, were also identified (Table S4).

We detected a total of 1,376 genes with copy number changes in our human lung cancer samples (File S1). Of these genes, 809 genes were amplified at a frequency of 37–66% at chromosome 7.

Based on this analysis we found an enrichment of genes involved in several cellular signaling pathways, particularly cell cycle control, cellular movement, cell death and embryonic development networks (data not shown), some of them listed in Table S4, suggesting that genetic and/or epigenetic aberrations are playing a functional important role in lung cancer biology.

### Increases in CNV at 7p21 and 7p15 Correlates with mRNA and Protein Overexpression and Not with DNA Methylation in Lung Cancer

**Figure 2:**
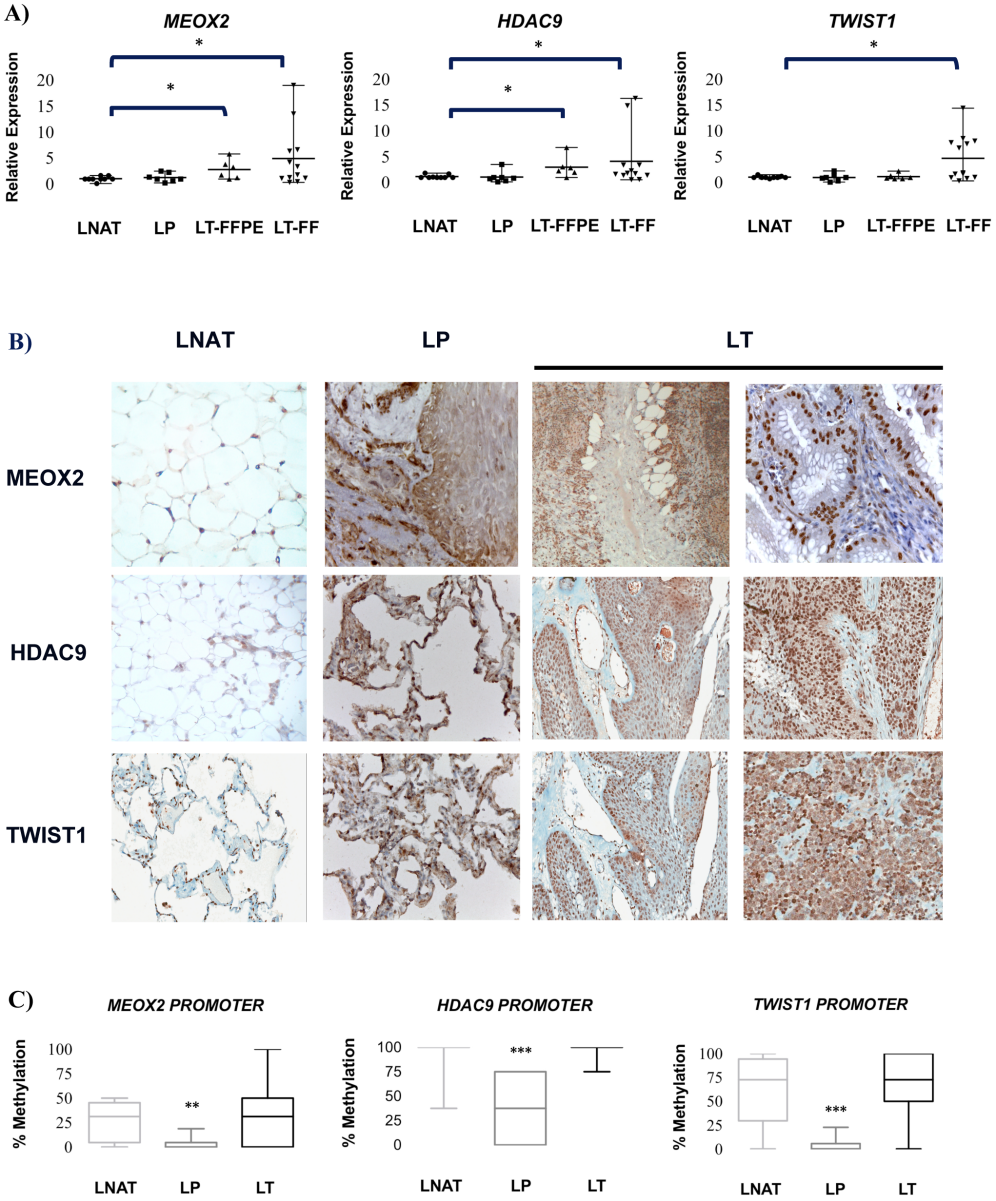
mRNA expression, DNA methylation and protein expression analyses in lung carcinomas. (A) *MEOX2*, *HDAC9* and *TWIST1* mRNA expression. Error bars represent 95% of confidence interval of the mean. (B) Protein expression in adjacent non-involved lung tissue (LNAT), lung precursor lesions (LP) and lung carcinomas (LT) (microphotographs at 200X and 400X), as well as formalin-fixed and paraffin-embedded (FFPE) and fresh frozen (FF) tissues, were compared. (C) Promoter sequence methylation analysis. The differences were statistically significant with respect to the LNAT and were detected using Fisher's exact test, an unpaired *t*-test and a Mann-Whitney U test, * *p*≤0.05; ** *p*≤0.005; *** *p*≤0.001. Error bars indicating min to max rank with 95% confidence interval, and box plots represent standard deviations of the mean.

The correlation between the increase in CNV and relative mRNA expression levels, as well as the impact of DNA methylation on mRNA expression profiles were analyzed in lung carcinomas. Using qRT-PCR assays, we found that only *MEOX2*, *HDAC9* and *TWIST1* (Figure 2A), as well as *AhR* and *EVX1* genes (Figure S3A–B) were over-expressed and positively correlated with CNV-high context (Figure 1B and C, and Figure S1), compared to other genes at 7p21 (not shown). The expression of these genes was significantly higher in the LT-derived samples than in the LNAT-derived samples (*p*≤0.05). In addition, the nuclear protein levels of *MEOX2*, *HDAC9* and *TWIST1* were consistently increased in the LT-derived samples (Figure 2B) as well as *MEOX2* and *TWIST1* protein levels/abundance in NSCLC cell lines (Figure S4A-C), whereas the expression of these proteins in the adjacent pulmonary parenchyma and the LNAT-derived samples were not increased (Figure 2B), as detected using immunohistochemistry. An increase in *EVX1* expression was detected at the membranes in the LP- and LT-derived samples and the NSCLC cell lines (Figure S5A–B).

To further explore the effect on gene expression of genes associated to CNV, the involvement of epigenetic changes, in particular DNA methylation was assessed. Methylation-sensitive enzymatic restriction assays revealed small differences in the DNA methylation percentage between the LNAT- and LT-derived samples in the promoter sequences of *MEOX2*, *HDAC9* and *TWIST1* (Figure 2C), suggesting a lack of correlation between mRNA expression and DNA methylation (Figure 2A and 2C). Additionally, we identified a reduction in DNA methylation for *MEOX2*, *HDAC9* and *TWIST1* (*p*≤0.005 and *p*≤0.001, respectively), in the LP-derived samples that were not observed in the LNAT- and LT-derived samples (Figure 2C).

As a complement, a paired analysis between the LNAT- and LT-derived samples in some CNV-free patients also revealed an mRNA overexpression of *MEOX2*, *HDAC9*, and *TWIST1*, suggesting a lack of genetic (CNV) and epigenetic (DNA methylation) correlation (Figure S6A-C), and a lack of genetic-epigenetic correlation in CNV-high patients (Figure S7A–C); with exception for the *AhR* gene between LNAT- and LT-samples (Figure S3A, S3C), and between CNV-free and CNV-high patients (S6D, and S7D). In summary, our data suggest that DNA methylation is not the main responsible for the observed expression patterns at the 7p21 cytogenetic region in lung carcinomas, such as NSCLC.

### Decreases of the Histone Code of H3K27me3 and Increases in H3K4me3 are Associated with *MEOX2* and *TWIST1* Overexpression and Correlated with Poor Clinical Prognosis in NSCLC

Therefore, we asked whether other epigenetic modifications might participate in the regulation of the affected genes, focusing at the 7p21 cytogenetic region in NSCLC.

On this, as a completely independent validation study, the role of histone modifications in the overexpression of *MEOX2* and *TWIST1* in NSCLC patients was evaluated. For this analysis an additional group of patients who had undergone a clinical follow-up was studied to determine whether *MEOX2* and *TWIST1* overexpression in the tumoral tissue was associated with the levels of H3K27me3 *vs.* H3K27Ac and H3K4me3 and might determine the prognosis and/or responsiveness to oncologic treatment in NSCLC patients.

**Figure 3:**
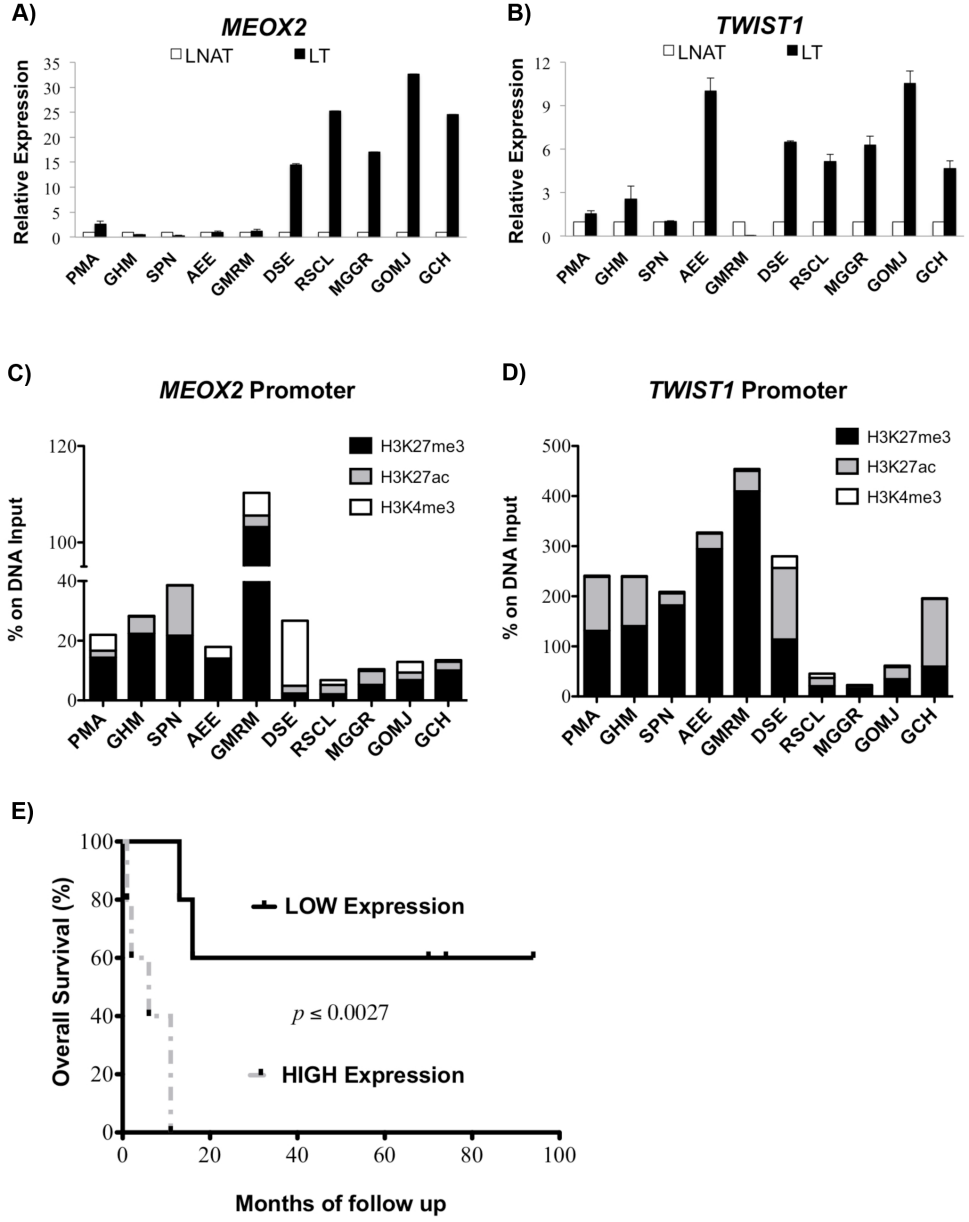
Analysis of mRNA expression, the histone code enrichment profiles of the *MEOX2* and *TWIST1* promoters and survival in NSCLC patients. (A) *MEOX2* expression in NSCLC and the LNAT-derived samples, and (B) *TWIST1* expression in NSCLC and the LNAT-derived samples. Error bars represent standard errors of the mean of three replicates. (C) Histone modification enrichment profile of the *MEOX2* promoter sequence. (D) Histone modification enrichment profile of the *TWIST1* promoter sequence. Box plots represent the mean of three replicates. The analysis revealed a correlation between low mRNA expression and H3K27me3 enrichment. (E) Survival curve analyses based on Log-Rank (Mantel Cox) and Gehan-Breslow-Wilcoxon tests of the relative expression index of *MEOX2* plus *TWIST1* (*p*≤0.0027).

A paired analysis between the LNAT- and LT-derived samples, in association with *MEOX2* and *TWIST1* overexpression (Figure 3A–B), revealed a reduction in H3K27me3 (Figure 3C–D). Those patients with lower enrichment levels of the repressive histone H3K27me3 and high *MEOX2* and *TWIST1* mRNA expression (Figure 3A–D) displayed poor survival rates, with an average of 6.2 months (DSE, RSCL, MGGR, GOMJ and GCH patients) whereas patients with higher H3K27me3 enrichment levels and low *MEOX2* and *TWIST1* mRNA expression (Figure 3A–D) displayed better survival rates, with an average of 53.4 months (GMRM, AEE, SPN, GHM and PMA patients, with *p*≤0.0027) (Figure 3E, Table 1).

**Table 1:** Outcomes clinical data of NSCLC patients.

Patient	Age	Gender	Wood Smoke	Tobacco/Aromatic Compounds	Family Heritage Cancer	Histological Type	TNM	Adjuvant Treatment (Cycles)/Response	Follow Up	Current Status	*MEOX2* Relative Expression Index	*TWIST1* Relative Expression Index
PMA	60	Female	Positive	Negative	Positive	AD	2,3,1	Cisplatinum- Vinorelbine (6)/Partial Response	94 Months	Alive	Low	Low
GHM	62	Female	Positive	Negative	Positive	AD	4,3,1	Cisplatinum- Vinorelbine (6)/Partial Response	70 Months	Alive	Low	Low
SPN	41	Female	Positive	Negative	Positive	AD	3,2,0	Cisplatinum- Vinorelbine (6)/Partial Response	74 Months	Alive	Low	Low
AEE	71	Female	Positive	Negative/Positive	Negative	AD	4,0,0	Carboplatinum- Gemcitabine (2)/Partial Response	16 Months	Dead	Low	High
GMRM	48	Female	Positive	Negative	Negative	AD	4,1,1	Cisplatinum- Docetaxel (6)/Partial Response	13 Months	Dead	Low	Low
DSE	70	Female	Negative	Positive/Negative	Negative	AD	4,3,1	Carboplatinum- Docetaxel (6)/No Response	11 Months	Dead	High	High
RSCL	36	Female	Negative	Negative	Negative	AD	4,2,1	Cisplatinum- Docetaxel (2)/No Response	11 Months	Dead	High	High
MGGR	55	Female	Negative	Positive/Negative	Negative	AD	N.D.	N.D.	6 Months	Dead	High	High
GOMJ	64	Female	Positive	Negative	Negative	AD	4,0,0	Cisplatinum (1)/Treatment Abandonment	2 Months	Dead	High	High
GCH	74	Female	Positive	Negative	Negative	AD	3,2,1	Patient Refuse Treatment/N.A.	1 Month	Dead	High	High

(AD) Adenocarcinoma Patients; (N.D.) Not Detected; and (N.A.) Not Applied.

**Figure 4:**
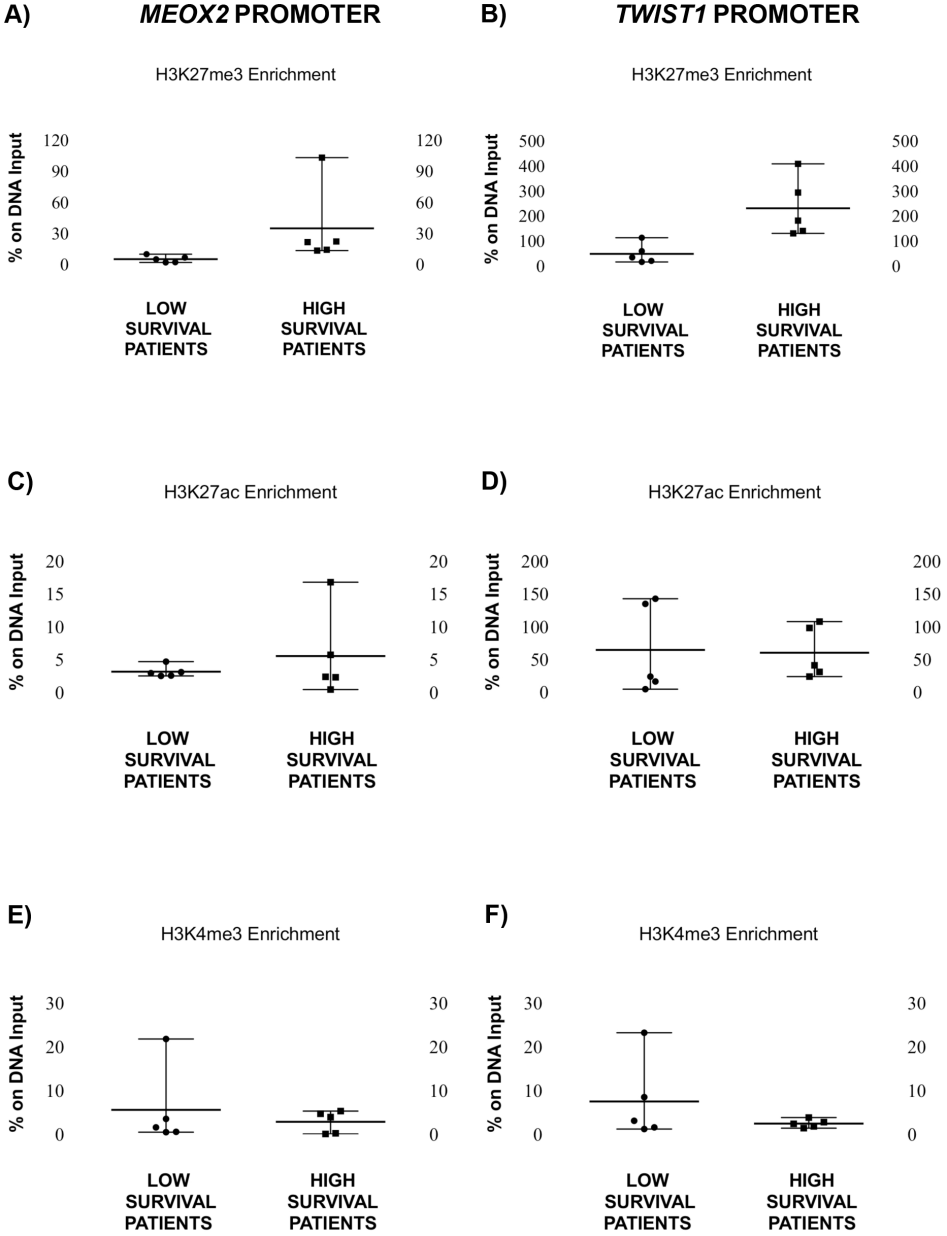
Analysis of the enrichment profile of H3K27me3, H3K27ac and H3K4me3 at the *MEOX2* and *TWIST1* promoters in NSCLC patients. (A) H3K27me3 enrichment in the *MEOX2* promoter sequence (**Mann-Whitney U test, *p*≤0.01, and F test, *p*≤0.0003) and (B) H3K27me3 enrichment in the *TWIST1* promoter sequence in high survival patients as compared to patients with poor prognoses (***Mann-Whitney U test, *p*≤0.01, and unpaired t-test, *p*≤0.02). (C) H3K27ac enrichment in the promoter sequence of *MEOX2* (no significant changes) and (D) H3K27ac enrichment in the promoter sequence of *TWIST1* in high survival patients compared to patients with poor prognoses (no significant changes). (E) H3K4me3 enrichment in the *MEOX2* promoter sequence (*F test, *p*≤0.02) and (F) the *TWIST1* promoter sequence in high survival patients compared to patients with poor prognoses (***F test, *p*≤0.0006). Error bars represent mean with range.

Also, significantly increased levels (*p*≤0.008) of the repressive histone marker H3K27me3 at both the *MEOX2* and *TWIST1* promoter sequences were confirmed for those patients with higher survival rates (Figure 4A–B). However, no significant changes in H3K27Ac levels were observed (Figure 4C–D). In contrast, reduced levels of the activation marker H3K4me3 were detected at both the *MEOX2* (*p*≤0.028) and *TWIST1* (*p*≤0.0006) promoter sequences in the high survival group of patients (Figure 4E–F) and were correlated with partial response to adjuvant cisplatinum or carboplatinum, as the first line chemotherapy in NSCLC patients (Table 1).

These findings suggest that decreased levels of the histone marker H3K27me3 and increased levels of H3K4me3 are correlated with *MEOX2* and *TWIST1* overexpression, probably associated to clinical response prognosis in NSCLC patients (Table 1), or involved in the acquisition of chemoresistance.

### Decreased Levels of H3K27me3 and Increased Levels of H3K4me3 Lead to Cisplatinum Failure in an *MEOX2*- and *TWIST1* -Dependent Manner in NSCLC

**Figure 5:**
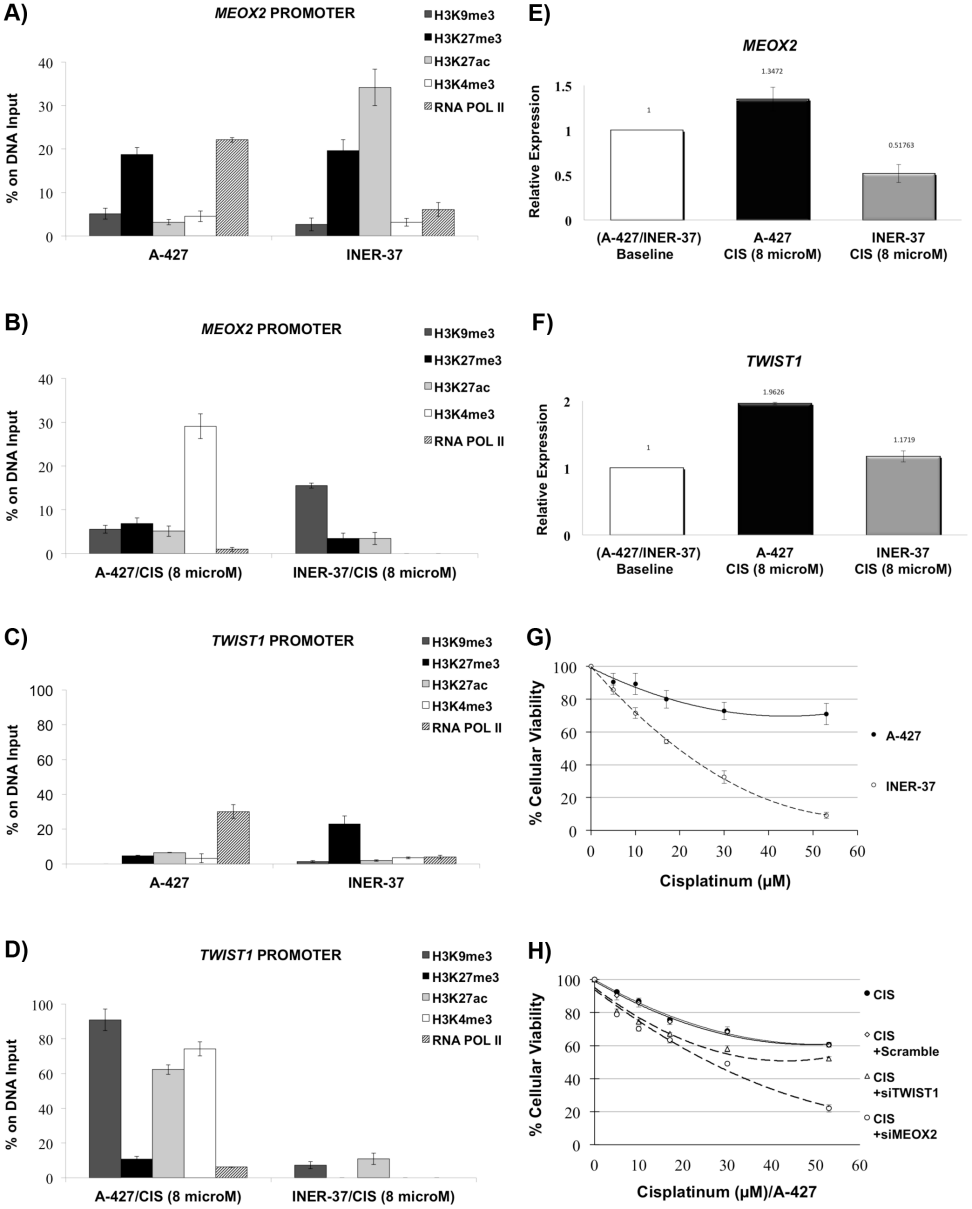
Histone modification profile at the *MEOX2* and *TWIST1* promoters under baseline conditions and after cisplatinum treatment in NSCLC cell lines (A-427 and INER-37). (A) *MEOX2* histone profile under baseline conditions. (B) *MEOX2* histone modification profile changes after cisplatinum treatment. (C) *TWIST1* histone profile under baseline conditions. (D) *TWIST1* histone modification profile changes after cisplatinum treatment. (E) *MEOX2* mRNA expression induction by cisplatinum stimulation as compared to the baseline conditions from both NSCLC cell lines. (F) *TWIST1* mRNA expression induction by cisplatinum stimulation as compared to the baseline conditions from both NSCLC cell lines. (G) Chemoresistance curves evaluating cellular viability for both NSCLC cell lines. (H) *MEOX2* and *TWIST1* siRNA silencing assays and cellular viability analyses. Statistically significant differences with respect to the baseline control conditions as assessed using an unpaired t-test, *p≤0.05 and **p≤0.001. Error bars represent standard errors of the mean of three technical replicates.

We detected a reduction in the rate of the repressive histone modification combination H3K27me3/H3K27Ac in the *MEOX2* promoter in both NSCLC cell lines INER-37 and A-427 (Figure 5A–B). In addition, an increase in the levels of the activation modification H3K4me3 was detected in A-427 cells (Figure 5B), whereas the repressive histone modification H3K9me3 was increased in INER-37 cells (Figure 5B). These results were correlated with a significant (*p*≤0.05) increase in *MEOX2* expression in A-427 cells and a significant (*p*≤0.001) decrease of *MEOX2* expression in INER-37 cells (Figure 5E).

The lower levels of H3K27me3 and the higher levels of H3K4me3 and H3K27Ac indicate activation of the expression of the *TWIST1* gene in A-427 cells (Figure 5C–D). In contrast, only marginal increases in H3K27Ac caused by cisplatinum exposure were observed in INER-37 cells (Figure 5C–D). As a result of these changes, *TWIST1* mRNA overexpression was induced in A-427 cells (*p*≤0.001) but not in INER-37 cells (Figure 5F).

Based on the results outlined above, we propose that *MEOX2* and *TWIST1* overexpression is associated with reduced H3K27me3 levels and that increased H3K4me3 levels at both the *MEOX2* and *TWIST1* promoters participate in resistance to oncologic drugs, as likely occurred in cisplatinum/carboplatinum-treated NSCLC patients (Table 1).

To test this hypothesis, cisplatinum-resistance assays were conducted in A-427 and INER-37 cells, revealing that A-427 cells developed greater chemoresistance than INER-37 cells (Figure 5G). According to this, we suggest that stochastic effects caused by cisplatinum reduced the levels of H3K27me3 and increased the levels of H3K4me3 at both the *MEOX2* and *TWIST1* sequence promoters (Figure 5B and D), promoting *MEOX2* and *TWIST1* mRNA overexpression (Figure 5E–F) and contributing to chemoresistance in A-427 cells (Figure 5G). These results were in accordance with the lack of induced mRNA expression in response to 5′-aza-deoxycytidine treatment; in contrast of the induction of mRNA expression using histone deacetylase inhibitor TSA (data not shown).

Finally, we demonstrated that cisplatinum chemoresistance is highly dependent upon *MEOX2* (rather than *TWIST1*) in the chemoresistant A-427 cell line using cellular cytotoxicity assays and genetic silencing (Figure 5H). In this regard, we observed that *MEOX2* protein expression is induced in a cisplatinum dose-dependent manner, where *MEOX2* inducible expression decreased by greater than 60% using siRNA/*MEOX2* silencing assays (data not shown). These results support our hypothesis that the loss of H3K27me3 and the enrichment of H3K4me3, as well as H3K27Ac in some cases (Figure 5A–D) exceed the presence or absence of CNVs at 7p21 inducing overexpression of *MEOX2* and *TWIST1*, associated to NSCLC patients prognosis.

## Discussion

Lung cancer has long been considered to affect primarily elderly people, but it has recently occurred with greater frequency in younger patients, suggesting the influence of external environmental factors that may supersede genetic susceptibility factors that are associated with lung cancer development, such as genetic polymorphisms at 5p15.33 [22], [23] and 6p21.33 [23].

Previously, chromosomal imbalances [7], [14] and high frequencies of CNV [10], [11], [12] on chromosomes 3, 5, 7 and 14, as well as epigenetic (methylated DNA) aberrations of *HOXA5*, *HOXA7* and *HOXA9* at 7p15.2, were associated with early clinical stage [17] and clinical progression [24] in NSCLC patients [17], [24] and NSCLC cell lines [14], [17]. As we demonstrated in the present study, high frequencies of CNV, histone H3K27 and H3K4 imbalances at the 7p21 locus also occur during the advanced clinical stages in NSCLC patients and NSCLC cell lines (Caucasian and Mexican Mestizo), and these features likely exist in lung precursor lesions and contribute to lung cancer progression, although further studies must be conducted to confirm these hypotheses.

However, reports have indicated that lung cancer development occurs preferentially through genomic imbalances. Other pathways, such as gains of chromosome 7 [16] and copy gains at the 5p and 8q subtelomeric regions, have been shown to contribute to the histopathological progression of bronchioloalveolar carcinomas to lung adenocarcinomas (AD). In particular, 7p genomic instability has been associated with aggressiveness and invasive lung tumors [25], and recurring gains at 7p21 (*e.g.*, *AGR2*) are often present in both lung AD cell lines and advanced NSCLC [26].

Our results suggest that *MEOX2*, *HDAC9*, *TWIST1*, *AhR* and *EVX1* overexpression from the 7p21 locus may be novel lung cancer or NSCLC tumor markers. This pattern of overexpression occurs in both patients with high frequencies of CNV and in those lacking CNV and is likely due to epigenetic aberrations that exceed the gains in CNV that are frequently reported at the 5p [12] and 7p loci [9], [10], [14], [26] in NSCLC, as have been previously described in the presence and absence of CNV at the 3p22.3 locus, but they are under the control of histone code aberrations in epithelial tumors [27]. These aberrations also likely occur for *IRX2* at the 5p15.33 locus [24], as well as *HOXA4* and *HOXA5* [27] and *HOXA7* plus *HOXA9* at the 7p15.2 locus [17]. And they may affect the functional behavior or clinical progression of NSCLC, either independent of or in addition to the main chromosomal disturbances previously described by The International Lung Cancer Consortium “ILCCO” [10].

Previous reports have indicated that increases in CNV in lung cancer are correlated with mRNA and protein overexpression. In contrast, overexpression is detected even in the absence of mutations, amplifications or CNVs, as has been described for other potential tumor markers including TFDP1 at the 13q34 locus [28] and *NKX2-8* [11] and *NKX2-1* [10] at the 14q13.3 locus, and as we demonstrated for the *MEOX2*, *HDAC9*, *TWIST1* and *AhR* genes at the 7p21 locus. Overexpression also occurs at the 1p31.1, 3p22.3, 5q12.1–5q13.2 and 7p15.2 chromosomal regions, which contain gains in CNV. However, at least in the case of the 3p22.3 locus, the regulation of mRNA expression appears to be under epigenetic control by the repressive histone marker H3K9me3, as detected using ChIp assays. This occurs even in the absence of high CNV and independent of DNA methylation status, as identified by the lack of expression in response to 5′-aza-deoxycytidine treatment [27], which has been described in different epithelial tumors [28], [27], but has been shown to be functionally involved in growth, survival and tumorigenic activity in NSCLC [10], [11], [28].

Our results revealed that *MEOX2* and *TWIST1* mRNA overexpression occurred in spite of the lack of significant differences in DNA methylation between the LNAT- and LT-derived samples, as previously described for *TWIST1* in NSCLC [29], as well as *MEOX2*, which displayed few differences in DNA methylation between fetal lung, normal adult lung and the LT-derived samples (0%, 20% and 40%, respectively), but was overexpressed in the LT-derived samples [30]. In addition, *MEOX2* promoter sequences were recently used as part of a test for cancer-specific DNA methylation cluster markers in colorectal cancer [31], and *TWIST1* methylation was assessed at promoter sequences in lung AD patients [32].

Supporting the idea that *MEOX2* and *TWIST1* contribute to oncologic treatment resistance, functional studies have shown that shRNA-mediated *TWIST1* silencing in cisplatinum-treated cervical cancer cells regulates the expression of the multi-drug resistance receptor, *MDR1*/*P-gp*, also known as *ABCB1* [33]. Other reports have described the segmental amplification of *ABCB1* at the 7q21.12 cytogenetic region in a NSCLC cell line that was resistant to paclitaxel compared to the NCI-H460 parental cell line [34]. Such cytogenetic amplification has been proposed to occur through the breakage fusion-bridge cycle model due to telomere attachment and the involvement of DNA repair mechanisms [35]. As likely occurred for CNVs at the 7p22.3–p22.1, 7p21.3–p21.1 and 7p15.3–p15.2 loci (present study), and as was previously reported for genomic amplifications at the 7p21.1 locus in taxol-resistant epithelial tumor cells, these results were likely dependent upon *TWIST1* overexpression [36]. Furthermore, *TWIST1* overexpression has been associated with poorer prognoses in resectable clinical stage I NSCLC patients [37].

In the present study, *MEOX2* overexpression was associated with chemoresistance in NSCLC, which was in contrast to its physiological functions in somite morphogenesis [38] and vascular endothelial growth arrest control via NFkB inhibition [39]. In addition, the DNA binding activity of *MEOX2* to the tumor suppressor *p16INKa* at the promoter level [40], [41] causes cell cycle arrest and vascular cell senescence [35], [36], [38].

Furthermore, we demonstrated that altered *MEOX2* and *TWIST1* expression in NSCLC is dependent upon the histone marker H3K27me3, which is catalyzed under normal conditions by the histone methyltransferase EZH2, a component of the Polycomb-repressive complex 2 (PRC2) [42]. However, in cancer cells, aberrations in *de novo* DNA methylation profiles and trimethylation of H3K27 [43] are associated with higher PRC2 occupancy at the cytogenetic regions for homeobox genes and DNA-binding proteins in NSCLC [44]. Such aberrations may also induce aberrant expression profiles, as we have described for *MEOX2* and *TWIST1* and as we suggest for *HDAC9*, *AhR* and *EVX1* at the 7p21 and 7p15 cytogenetic regions that are controlled by loss of histone trimethylation and participate in the progression and prognosis of NSCLC patients [45].

Our data suggest that cisplatinum drug administration modifies the balance of the histone code of H3K27me3 *vs.* H3K27Ac toward a significantly lower enrichment of H3K27me3 and a significantly higher enrichment of H3K4me3 at both the *MEOX2* and *TWIST1* promoter sequences, which is likely in accordance with the disruption of the nucleosome, histone translocations and RNA Pol failure function, as was previously reported to be caused by cisplatinum [46]. However, prior to the present study, *MEOX2* and *TWIST1* overexpression, which are under the epigenetic control of H3K27me3, were not known to be involved in chemoresistance and poor prognosis in NSCLC, although EZH2-mediated trimethylation of H3K27 has been previously correlated with bronchoepithelial oncogenic transformation and evasion of apoptosis [47]. These results strongly support the idea that histone code imbalances, such as H3K9Ac *vs.* H3K4me [48] and H3K27me3 [47], can exceeds CNV gains and may determine clinical progression and poor prognosis in NSCLC patients.

In this context, our results also suggest that *HDAC9* overexpression likely contributes to histone profile aberrations, which have not been previously described to play a role in NSCLC progression or chemoresistance. However, *HDAC9* was recently described as a potential tumor suppressor gene that is involved in cell growth control in human immortalized lung epithelial cells and NSCLC cell lines *in vitro* [49]. In contrast to these results, *HDAC9* has been described overexpressed in NSCLC patients, as observed in the present study and as reported by Okudela et al. in some NSCLC patients, and thus, *HDAC9* is likely involved in lung carcinogenesis [49].

In summary, we demonstrated for the first time that *MEOX2* is involved in NSCLC clinical progression and chemoresistance, but the mechanisms involved remain uncharacterized. Nonetheless, our findings can likely be explained by xenobiotic or oncologic drug detoxification pathways that are mediated by *glutathione-S-transferase M4* (*GSTM4*) and/or *glutaredoxin* (*thiol transferase*; *GLRX*), both of which are overexpressed by the transcription factor *MEOX2* [39]. Also, single nucleotide polymorphisms (SNPs) in *MEOX2* are also strongly associated with clinical risk progression in NSCLC patients [50].

Finally, we demonstrated chemoresistance in NSCLC in a *MEOX2* and *TWIST1* overexpression-dependent manner, both in the absence or presence of high CNV. Thus, we hypothesize that epigenetic aberrations cause imbalances in the H3K27me3/Ac and H3K4me3 histone code at the 7p21 locus and that epigenetic mechanisms can exceed CNV, associated probably to poor survival and promoting resistance to oncologic drug treatment such as cisplatinum and potentially contributing to the poor survival in NSCLC patients. However additional clinical studies must be performed to confirm our findings, as new probable clinical markers of follow-up in NSCLC patients.

## Supporting Information

Figure S1 **Genome wide focal analysis at chromosome 7.** Focal analysis of chromosome 7 from lung normal adjacent tissues (Processing as FF), lung precursor and lung tumor samples (Processing as FFPE) (CNV in 13 of 14 lung lesion samples); consistent chromosomal amplification is shows in 7p21 (Arrow).
(TIF)

Figure S2 **Whole genome analysis.** Genome wide comparation analysis on lung precursor (LP) lesion *versus* lung carcinoma (LT), using SNP 500 K and SNP 6.0 platforms. Arrows indicates CNV at 7p, using both array-platforms.
(TIF)

Figure S3 **mRNA expression and DNA promoter methylation analysis.** (A) AhR mRNA Expression Profile, Fisher exact test with * *p*≤0.05. (B) EVX1 mRNA expression profile, Unpaired t test and Mann-Whitney U test, with ** *p*≤0.05. (C) AhR promoter methylation analysis. Statistically differences by Fisher exact test, Unpaired t test and Mann-Whitney U test, with *** *p*≤0.005; are indicated with respect to Lung Normal Adjacent Tissues (LNAT). LNAT and LT matched tissues were analyzed. DNA methylation status was not possible detected on *EVX1* promoter.
(TIF)

Figure S4 **MEOX2 and TWIST1 protein expression analysis in Non-Small Cell Lung Carcinoma cell lines.** (A) Immuno-histochemical Protein MEOX2 expression, (B) Immuno-histochemical Protein TWIST1 expression (Microphotographs at 200X and 400X), and (C) Western Blot Protein MEOX2 and TWIST1 expression in NSCLC cell lines (A-549, A-427) from ATCC; and (INER-51, INER-37) established from Mestizo Mexican patients.
(TIF)

Figure S5 **EVX1 protein expression analysis in Lung Normal and Lung Lesion Tissues, as well Non-Small Cell Lung Carcinoma cell lines.** (A) EVX1 protein expression in lung normal adjacent, lung precursor and lung carcinoma lesions. Using the Formalin Fixed and Paraffin Embedded (FFPE) method. (B) EVX1 protein expression in NSCLC cell lines A-549, A-427, INER-51, and INER-37 (Microphotographs at 200X and 400X).
(TIF)

Figure S6 **Promoter methylation and mRNA expression paired analysis for no-change CNV patients.** Correlation analysis between lung normal adjacent to lung tumor (LNAT), and lung tumor (LT). (A) *MEOX2*, (B) *HDAC9*, (C) *TWIST1*, and (D) *AhR* for patients without change CNV.
(TIF)

Figure S7 **Promoter methylation and mRNA expression paired analysis for high-CNV patients.** Correlation analysis between lung normal adjacent to lung tumor (LNAT), and lung tumor (LT). (A) *MEOX2*, (B) *HDAC9*, (C) *TWIST1*, and (D) *AhR* for patients with high frequency of CNV.
(TIF)

Table S1 **Oligonucleotide sequences for mRNA expression assays.**
(DOC)

Table S2 **Oligonucleotide sequences for promoter analysis.**
(DOC)

Table S3 **Restriction sites and combination of restriction enzymes.**
(DOC)

Table S4 **High frequency micro-aberrations detected at 7p in lung cancer patients.**
(DOC)

File S1 **Total copy number changes -amplifications and deletions- detected at complete genome and chromosome 7 in lung cancer samples.**
(XLSX)

## References

[1] Siegel R, Naishadham D, Jemal A. (2013) Cancer statistics, 2013. CA Cancer J Clin 63:11–30. 23335087 10.3322/caac.21166

[2] Sun S, Schiller JH, Gazdar AF. (2007) Lung cancer in never smokers—a different disease. Nat Rev Cancer 7:778–790. 17882278 10.1038/nrc2190

[3] Jemal A, Tiwari RC, Murray T, Ghafoor A, Samuels A, et al (2004) Cancer statistics, 2004. CA Cancer J Clin 54:8–29. 14974761 10.3322/canjclin.54.1.8

[4] Jemal A, Siegel R, Ward E, Hao Y, Xu J, et al (2008) Cancer statistics, 2008. CA Cancer J Clin 58:71–96. 18287387 10.3322/CA.2007.0010

[5] Jemal A, Bray F, Center MM, Ferlay J, Ward E, et al (2011) Global cancer statistics. CA Cancer J Clin 61:69–90. 21296855 10.3322/caac.20107

[6] Luk C, Tsao MS, Bayani J, Shepherd F, Squire JA. (2001) Molecular cytogenetic analysis of non-small cell lung carcinoma by spectral karyotyping and comparative genomic hybridization. Cancer Genet Cytogenet 125:87–99. 11369051 10.1016/s0165-4608(00)00363-0

[7] Petersen I, Bujard M, Petersen S, Wolf G, Goeze A, et al (1997) Patterns of chromosomal imbalances in adenocarcinoma and squamous cell carcinoma of the lung. Cancer Res 57:2331–2335. 9192802

[8] Björkqvist A-M, Husgafvel-Pursiainen K, Anttila S, Karjalainen A, Tammilehto L, et al (1998) DNA gains in 3q occur frequently in squamous cell carcinoma of the lung, but not in adenocarcinoma. Genes Chromosomes Cancer 22:79–82. 9591638

[9] Pei J, Balsara BR, Li W, Litwin S, Gabrielson E, et al (2001) Genomic imbalances in human lung adenocarcinomas and squamous cell carcinomas. Genes Chromosomes Cancer 31:282–287. 11391799 10.1002/gcc.1145

[10] Weir BA, Woo MS, Getz G, Perner S, Ding L, et al (2007) Characterizing the cancer genome in lung adenocarcinoma. Nature 450:893–898. 17982442 10.1038/nature06358 PMC2538683

[11] Harris T, Pan Q, Sironi J, Lutz D, Tian J, et al (2011) Both gene amplification and allelic loss occur at 14q13.3 in lung cancer. Clin Cancer Res 17:690–699. 21148747 10.1158/1078-0432.CCR-10-1892 PMC3041868

[12] Garnis C, Davies JJ, Buys TP, Tsao MS, MacAulay C, et al (2005) Chromosome 5p aberrations are early events in lung cancer: implication of glial cell line-derived neurotrophic factor in disease progression. Oncogene 24:4806–4812. 15870700 10.1038/sj.onc.1208643

[13] Lau SK, Boutros PC, Pintilie M, Blackhall FH, Zhu CQ, et al (2007) Three-gene prognostic classifier for early-stage non small-cell lung cancer. J Clin Oncol 25:5562–5569. 18065728 10.1200/JCO.2007.12.0352

[14] Campbell JM, Lockwood WW, Buys TP, Chari R, Coe BP, et al (2008) Integrative genomic and gene expression analysis of chromosome 7 identified novel oncogene loci in non-small cell lung cancer. Genome 51:1032–1039. 19088816 10.1139/G08-086

[15] Ubagai T, Matsuura S, Tauchi H, Itou K, Komatsu K. (2001) Comparative genomic hybridization analysis suggests a gain of chromosome 7p associated with lymph node metastasis in non-small cell lung cancer. Oncol Rep 8:83–88. 11115574 10.3892/or.8.1.83

[16] Hoglund M, Gisselsson D, Hansen GB, Mitelman F. (2004) Statistical dissection of cytogenetic patterns in lung cancer reveals multiple modes of karyotypic evolution independent of histological classification. Cancer Genet Cytogenet 154:99–109. 15474144 10.1016/j.cancergencyto.2004.01.030

[17] Rauch T, Wang Z, Zhang X, Zhong X, Wu X, et al (2007) Homeobox gene methylation in lung cancer studied by genome-wide analysis with a microarray-based methylated CpG island recovery assay. Proc Natl Acad Sci U S A 104:5527–5532. 17369352 10.1073/pnas.0701059104 PMC1838508

[18] de Lucio B, Manuel V, Barrera-Rodriguez R. (2005) Characterization of human NSCLC cell line with innate etoposide-resistance mediated by cytoplasmic localization of topoisomerase II alpha. Cancer Sci 96:774–783. 16271071 10.1111/j.1349-7006.2005.00111.x PMC11158927

[19] Ponce de Leon V, Barrera-Rodriguez R. (2005) Changes in P-glycoprotein activity are mediated by the growth of a tumour cell line as multicellular spheroids. Cancer Cell Int 5:20. 16001980 10.1186/1475-2867-5-20 PMC1185553

[20] Gorringe KL, Jacobs S, Thompson ER, Sridhar A, Qiu W, et al (2007) High-resolution single nucleotide polymorphism array analysis of epithelial ovarian cancer reveals numerous microdeletions and amplifications. Clin Cancer Res 13:4731–4739. 17699850 10.1158/1078-0432.CCR-07-0502

[21] Silva-Zolezzi I, Hidalgo-Miranda A, Estrada-Gil J, Fernandez-Lopez JC, Uribe-Figueroa L, et al (2009) Analysis of genomic diversity in Mexican Mestizo populations to develop genomic medicine in Mexico. Proc Natl Acad Sci U S A 106:8611–8616. 19433783 10.1073/pnas.0903045106 PMC2680428

[22] McKay JD, Hung RJ, Gaborieau V, Boffetta P, Chabrier A, et al (2008) Lung cancer susceptibility locus at 5p15.33. Nat Genet 40:1404–1406. 18978790 10.1038/ng.254 PMC2748187

[23] Wang Y, Broderick P, Webb E, Wu X, Vijayakrishnan J, et al (2008) Common 5p15.33 and 6p21.33 variants influence lung cancer risk. Nat Genet 40:1407–1409. 18978787 10.1038/ng.273 PMC2695928

[24] Rauch TA, Zhong X, Wu X, Wang M, Kernstine KH, et al (2008) High-resolution mapping of DNA hypermethylation and hypomethylation in lung cancer. Proc Natl Acad Sci U S A 105:252–257. 18162535 10.1073/pnas.0710735105 PMC2224196

[25] Aviel-Ronen S, Coe BP, Lau SK, da Cunha Santos G, Zhu CQ, et al (2008) Genomic markers for malignant progression in pulmonary adenocarcinoma with bronchioloalveolar features. Proc Natl Acad Sci U S A 105:10155–10160. 18632575 10.1073/pnas.0709618105 PMC2465804

[26] Zhu H, Lam DC, Han KC, Tin VP, Suen WS, et al (2007) High resolution analysis of genomic aberrations by metaphase and array comparative genomic hybridization identifies candidate tumour genes in lung cancer cell lines. Cancer Lett 245:303–314. 16517066 10.1016/j.canlet.2006.01.020

[27] Stransky N, Vallot C, Reyal F, Bernard-Pierrot I, de Medina SG, et al (2006) Regional copy number-independent deregulation of transcription in cancer. Nat Genet 38:1386–1396. 17099711 10.1038/ng1923

[28] Castillo SD, Angulo B, Suarez-Gauthier A, Melchor L, Medina PP, et al (2010) Gene amplification of the transcription factor DP1 and CTNND1 in human lung cancer. J Pathol 222:89–98. 20556744 10.1002/path.2732

[29] Anglim PP, Galler JS, Koss MN, Hagen JA, Turla S, et al (2008) Identification of a panel of sensitive and specific DNA methylation markers for squamous cell lung cancer. Mol Cancer 7:62. 18616821 10.1186/1476-4598-7-62 PMC2483990

[30] Cortese R, Hartmann O, Berlin K, Eckhardt F. (2008) Correlative gene expression and DNA methylation profiling in lung development nominate new biomarkers in lung cancer. Int J Biochem Cell Biol 40:1494–1508. 18203646 10.1016/j.biocel.2007.11.018

[31] De Carvalho DD, Sharma S, You JS, Su SF, Taberlay PC, et al (2012) DNA methylation screening identifies driver epigenetic events of cancer cell survival. Cancer Cell 21:655–667. 22624715 10.1016/j.ccr.2012.03.045 PMC3395886

[32] Tsou JA, Galler JS, Siegmund KD, Laird PW, Turla S, et al (2007) Identification of a panel of sensitive and specific DNA methylation markers for lung adenocarcinoma. Mol Cancer 6:70. 17967182 10.1186/1476-4598-6-70 PMC2206053

[33] Zhu K, Chen L, Han X, Wang J, Wang J. (2012) Short hairpin RNA targeting Twist1 suppresses cell proliferation and improves chemosensitivity to cisplatin in HeLa human cervical cancer cells. Oncol Rep 27:1027–1034. 22245869 10.3892/or.2012.1633 PMC3583405

[34] Kitada K, Yamasaki T. (2007) The MDR1/ABCB1 regional amplification in large inverted repeats with asymmetric sequences and microhomologies at the junction sites. Cancer Genet Cytogenet 178:120–127. 17954267 10.1016/j.cancergencyto.2007.06.014

[35] Kitada K, Yamasaki T. (2008) The complicated copy number alterations in chromosome 7 of a lung cancer cell line is explained by a model based on repeated breakage-fusion-bridge cycles. Cancer Genet Cytogenet 185:11–19. 18656688 10.1016/j.cancergencyto.2008.04.005

[36] Wang X, Ling MT, Guan XY, Tsao SW, Cheung HW, et al (2004) Identification of a novel function of TWIST, a bHLH protein, in the development of acquired taxol resistance in human cancer cells. Oncogene 23:474–482. 14724576 10.1038/sj.onc.1207128

[37] Jiang W, Pang XG, Wang Q, Shen YX, Chen XK, et al (2012) Prognostic role of Twist, Slug, and Foxc2 expression in stage I non-small-cell lung cancer after curative resection. Clin Lung Cancer 13:280–287. 22178381 10.1016/j.cllc.2011.11.005

[38] Mankoo BS, Skuntz S, Harrigan I, Grigorieva E, Candia A, et al (2003) The concerted action of Meox homeobox genes is required upstream of genetic pathways essential for the formation, patterning and differentiation of somites. Development 130:4655–4664. 12925591 10.1242/dev.00687

[39] Patel S, Leal AD, Gorski DH. (2005) The homeobox gene Gax inhibits angiogenesis through inhibition of nuclear factor-kappaB-dependent endothelial cell gene expression. Cancer Res 65:1414–1424. 15735029 10.1158/0008-5472.CAN-04-3431

[40] Irelan JT, Gutierrez Del Arroyo A, Gutierrez A, Peters G, Quon KC, et al (2009) A functional screen for regulators of CKDN2A reveals MEOX2 as a transcriptional activator of INK4a. PLoS One 4:e5067. 19340300 10.1371/journal.pone.0005067 PMC2659797

[41] Douville JM, Cheung DY, Herbert KL, Moffatt T, Wigle JT. (2011) Mechanisms of MEOX1 and MEOX2 regulation of the cyclin dependent kinase inhibitors p21 and p16 in vascular endothelial cells. PLoS One 6:e29099. 22206000 10.1371/journal.pone.0029099 PMC3243699

[42] Lee TI, Jenner RG, Boyer LA, Guenther MG, Levine SS, et al (2006) Control of developmental regulators by Polycomb in human embryonic stem cells. Cell 125:301–313. 16630818 10.1016/j.cell.2006.02.043 PMC3773330

[43] Schlesinger Y, Straussman R, Keshet I, Farkash S, Hecht M, et al (2007) Polycomb-mediated methylation on Lys27 of histone H3 pre-marks genes for de novo methylation in cancer. Nat Genet 39:232–236. 17200670 10.1038/ng1950

[44] McCabe MT, Lee EK, Vertino PM. (2009) A multifactorial signature of DNA sequence and polycomb binding predicts aberrant CpG island methylation. Cancer Res 69:282–291. 19118013 10.1158/0008-5472.CAN-08-3274 PMC2653261

[45] Van Den Broeck A, Brambilla E, Moro-Sibilot D, Lantuejoul S, Brambilla C, et al (2008) Loss of histone H4K20 trimethylation occurs in preneoplasia and influences prognosis of non-small cell lung cancer. Clin Cancer Res 14:7237–7245. 18974389 10.1158/1078-0432.CCR-08-0869

[46] Todd RC, Lippard SJ. (2010) Consequences of cisplatin binding on nucleosome structure and dynamics. Chem Biol 17:1334–1343. 21168769 10.1016/j.chembiol.2010.10.018 PMC3008157

[47] Watanabe H, Soejima K, Yasuda H, Kawada I, Nakachi I, et al (2008) Deregulation of histone lysine methyltransferases contributes to oncogenic transformation of human bronchoepithelial cells. Cancer Cell Int 8:15. 18980680 10.1186/1475-2867-8-15 PMC2584620

[48] Barlesi F, Giaccone G, Gallegos-Ruiz MI, Loundou A, Span SW, et al (2007) Global histone modifications predict prognosis of resected non small-cell lung cancer. J Clin Oncol 25:4358–4364. 17906200 10.1200/JCO.2007.11.2599

[49] Okudela K, Mitsui H, Suzuki T, Woo T, Tateishi Y, et al (2014) Expression of HDAC9 in lung cancer - potential role in lung carcinogenesis. Int J Clin Exp Pathol 7:213–220. 24427341 PMC3885475

[50] Frullanti E, Galvan A, Falvella FS, Manenti G, Colombo F, et al (2011) Multiple genetic loci modulate lung adenocarcinoma clinical staging. Clin Cancer Res 17:2410–2416. 21242121 10.1158/1078-0432.CCR-10-2394

