# Correction: Overexpression of *MEOX2* and *TWIST1* is Associated with H3K27me3 Levels and Determines Lung Cancer Chemoresistance and Prognosis

**DOI:** 10.1371/journal.pone.0146569

**Published:** 2016-02-10

**Authors:** Federico Ávila-Moreno, Leonel Armas-López, Aldo M. Álvarez-Moran, Zoila López-Bujanda, Blanca Ortiz-Quintero, Alfredo Hidalgo-Miranda, Francisco Urrea-Ramírez, R. María Rivera-Rosales, Eugenia Vázquez-Manríquez, Erika Peña-Mirabal, José Morales-Gómez, Juan C. Vázquez-Minero, José L. Téllez-Becerra, Roberto Ramírez-Mendoza, Alejandro Ávalos-Bracho, Enrique Guzmán de Alba, Karla Vázquez-Santillán, Vilma Maldonado-Lagunas, Patricio Santillán-Doherty, Patricia Piña-Sánchez, Joaquin Zúñiga-Ramos

In Fig 4, the statistical significance indicators are missing. Please see the corrected Fig 4 here.

**Fig 4:**
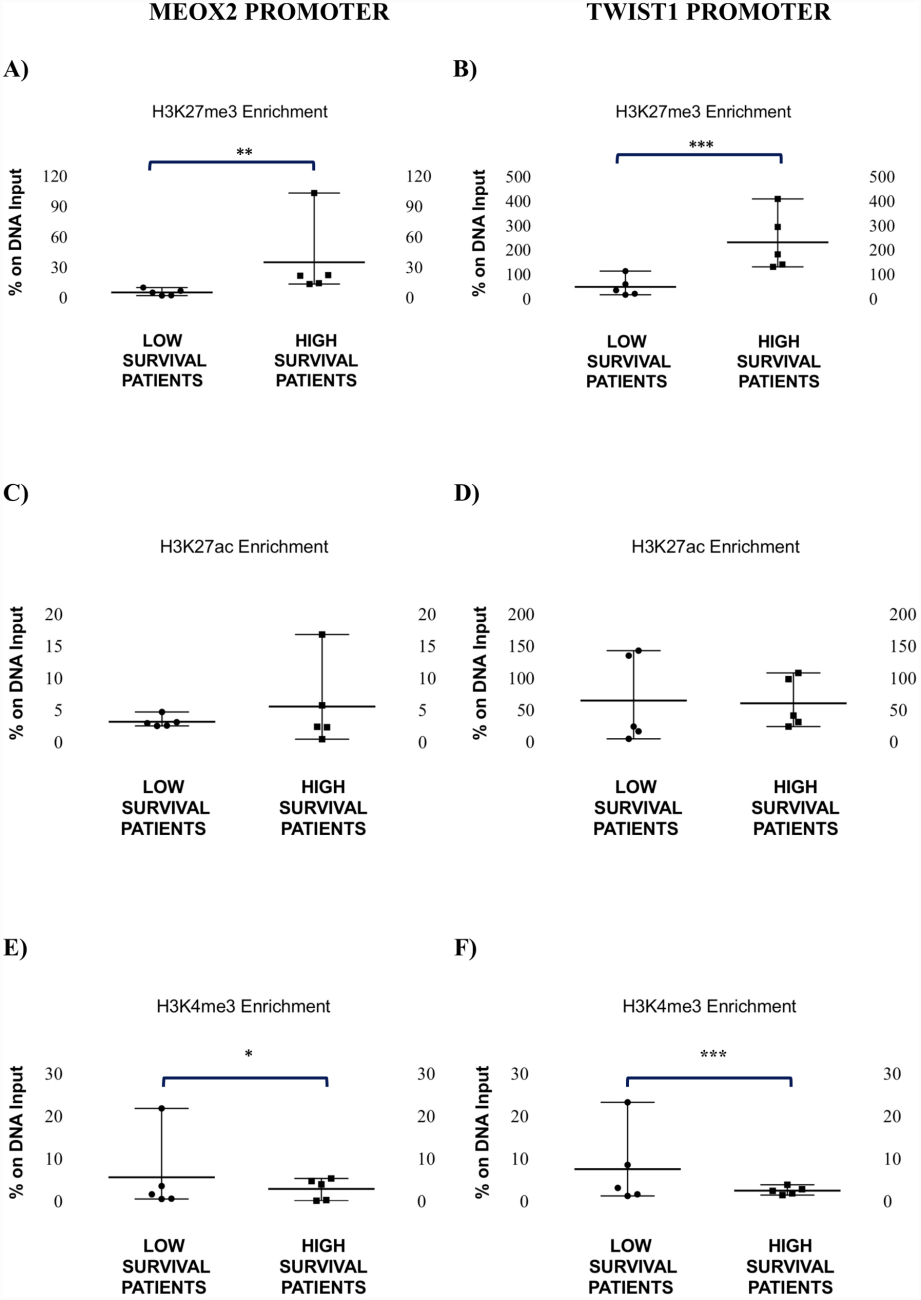
Analysis of the enrichment profile of H3K27me3, H3K27ac and H3K4me3 at the *MEOX2* and *TWIST1* promoters in NSCLC patients. (A) H3K27me3 enrichment in the *MEOX2* promoter sequence (**Mann-Whitney U test, *p*≤0.01, and F test, *p*≤0.0003) and (B) H3K27me3 enrichment in the *TWIST1* promoter sequence in high survival patients as compared to patients with poor prognoses (***Mann-Whitney U test,*p*≤0.01, and unpaired t-test, *p*≤0.02). (C) H3K27ac enrichment in the promoter sequence of *MEOX2* (no significant changes) and (D) H3K27ac enrichment in the promoter sequence of *TWIST1* in high survival patients compared to patients with poor prognoses (no significant changes). (E) H3K4me3 enrichment in the *MEOX2* promoter sequence (*F test, *p*≤0.02) and (F) the *TWIST1* promoter sequence in high survival patients compared to patients with poor prognoses (***F test, *p*≤0.0006). Error bars represent mean with range.

## References

[1] Ávila-Moreno F, Armas-López L, Álvarez-Moran AM, López-Bujanda Z, Ortiz-Quintero B, Hidalgo-Miranda A, et al (2014) Overexpression of *MEOX2* and *TWIST1* Is Associated with H3K27me3 Levels and Determines Lung Cancer Chemoresistance and Prognosis. PLoS ONE 9(12): e114104 doi: 10.1371/journal.pone.0114104 25460568 10.1371/journal.pone.0114104 PMC4252097

