# Supplementary figures and images for: Overexpression of MEOX2 and TWIST1 Is Associated with H3K27me3 Levels and Determines Lung Cancer Chemoresistance and Prognosis
Source: PLoS One. 2014 Dec 2;9(12):e114104. doi: 10.1371/journal.pone.0114104 (PMC4252097; doi:10.1371/journal.pone.0114104)

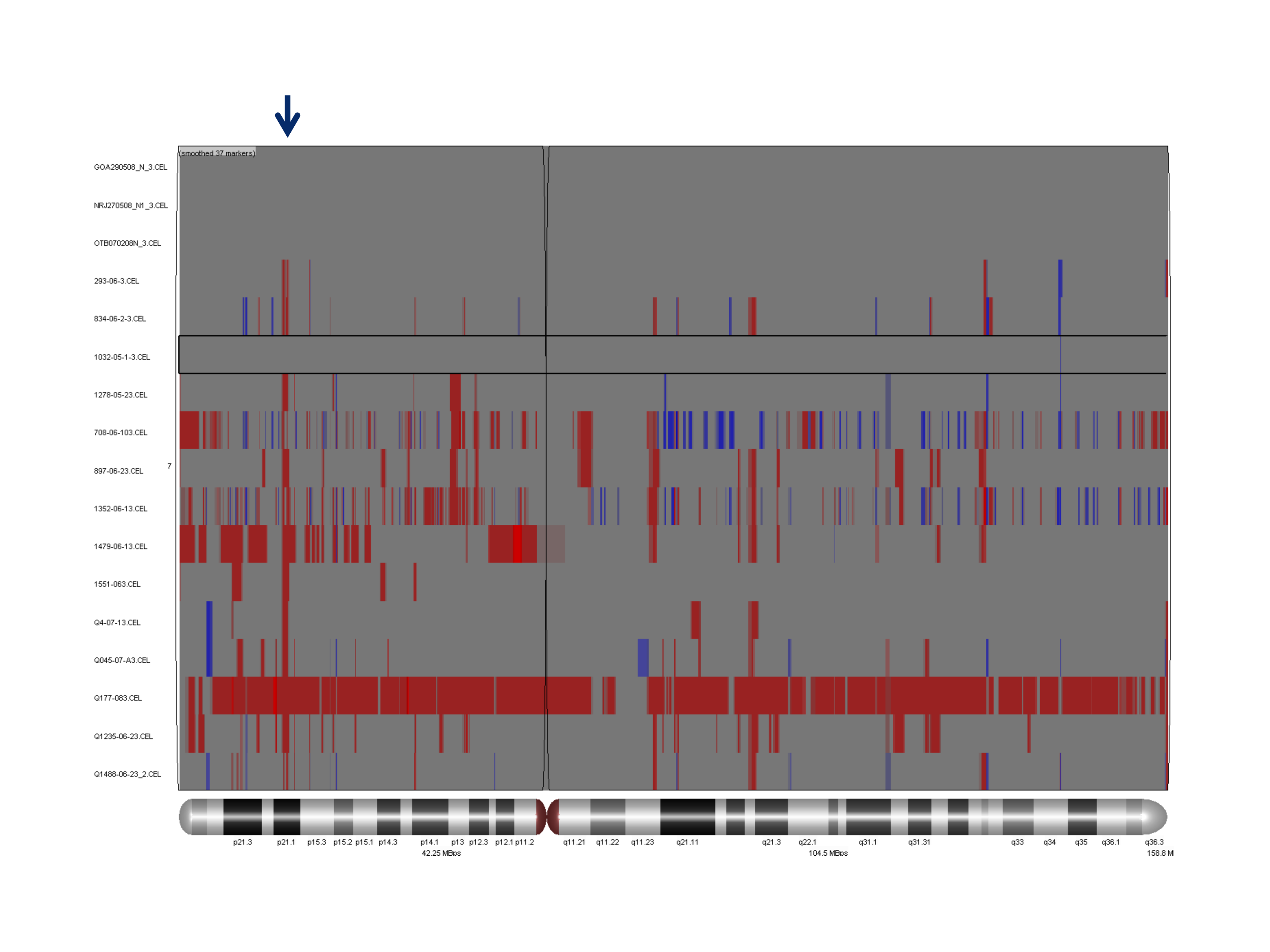

Supplement: Figure S1 — Genome wide focal analysis at chromosome 7. Focal analysis of chromosome 7 from lung normal adjacent tissues (Processing as FF), lung precursor and lung tumor samples (Processing as FFPE) (CNV in 13 of 14 lung lesion samples); consistent chromosomal amplification is shows in 7p21 (Arrow). (TIF) [file pone.0114104.s001.tif]

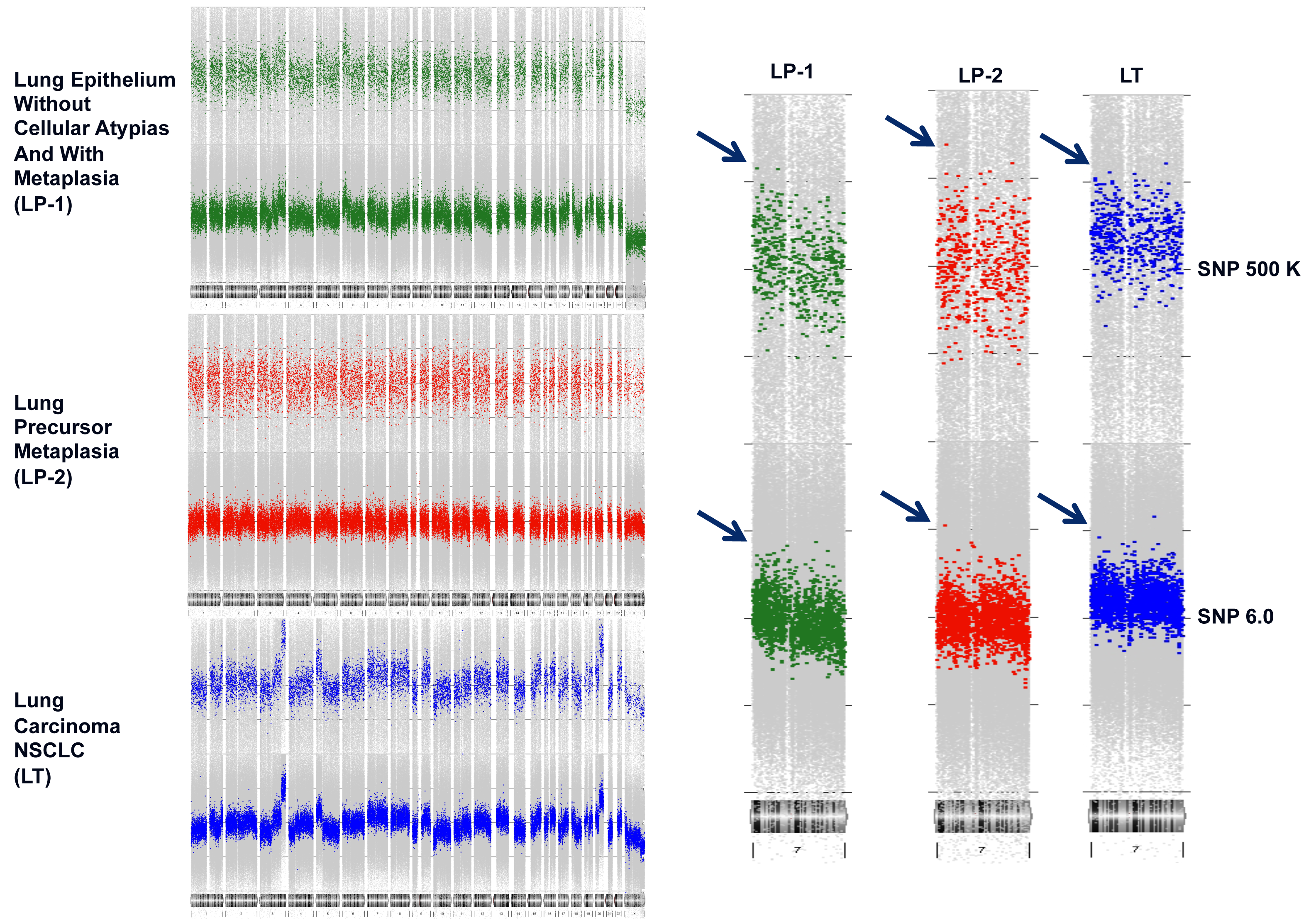

Supplement: Figure S2 — Whole genome analysis. Genome wide comparation analysis on lung precursor (LP) lesion versus lung carcinoma (LT), using SNP 500 K and SNP 6.0 platforms. Arrows indicates CNV at 7p, using both array-platforms. (TIF) [file pone.0114104.s002.tif]

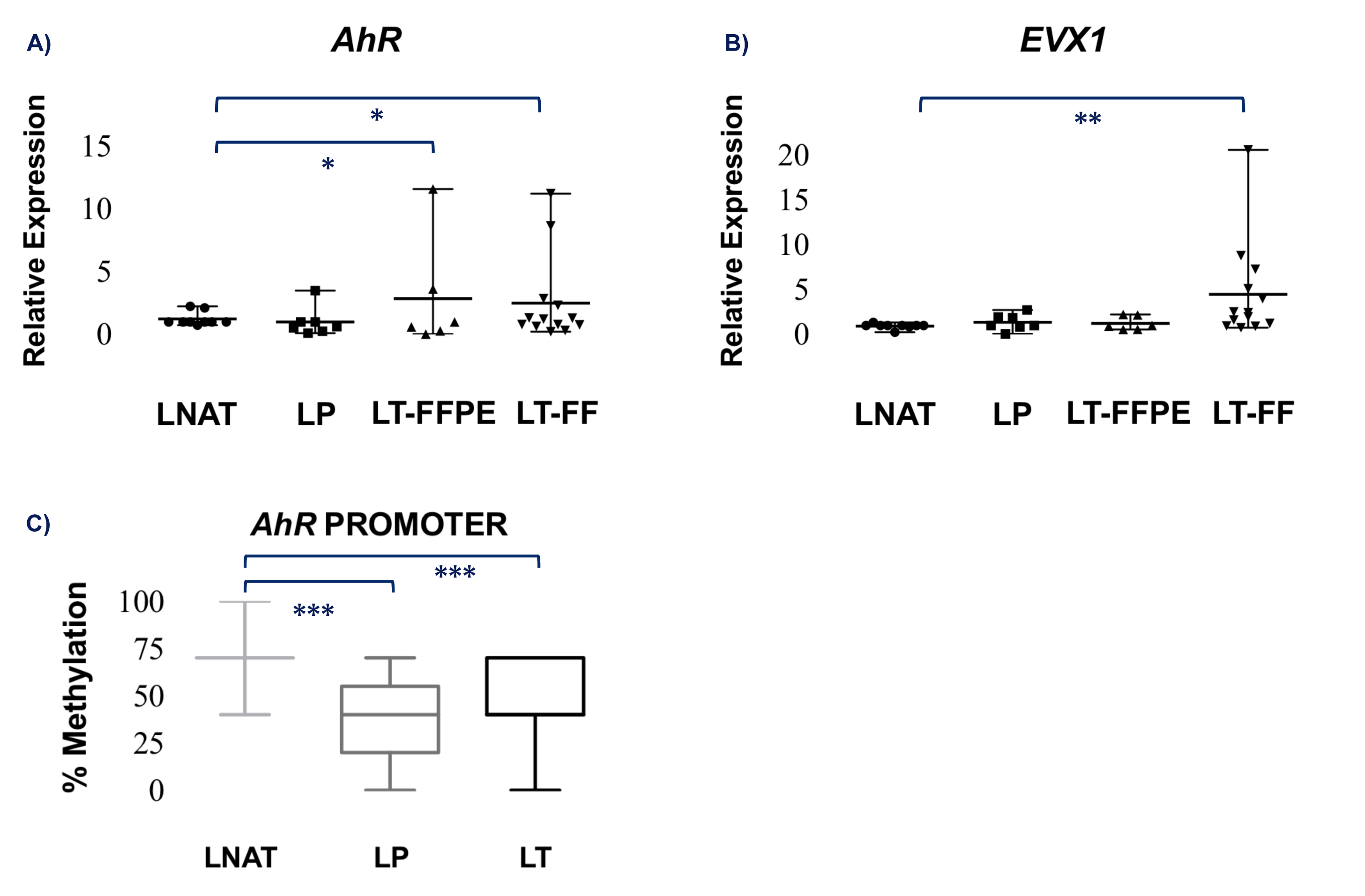

Supplement: Figure S3 — mRNA expression and DNA promoter methylation analysis. (A) AhR mRNA Expression Profile, Fisher exact test with * p≤0.05. (B) EVX1 mRNA expression profile, Unpaired t test and Mann-Whitney U test, with ** p≤0.05. (C) AhR promoter methylation analysis. Statistically differences by Fisher exact test, Unpaired t test and Mann-Whitney U test, with *** p≤0.005; are indicated with respect to Lung Normal Adjacent Tissues (LNAT). LNAT and LT matched tissues were analyzed. DNA methylation status was not possible detected on EVX1 promoter. (TIF) [file pone.0114104.s003.tif]

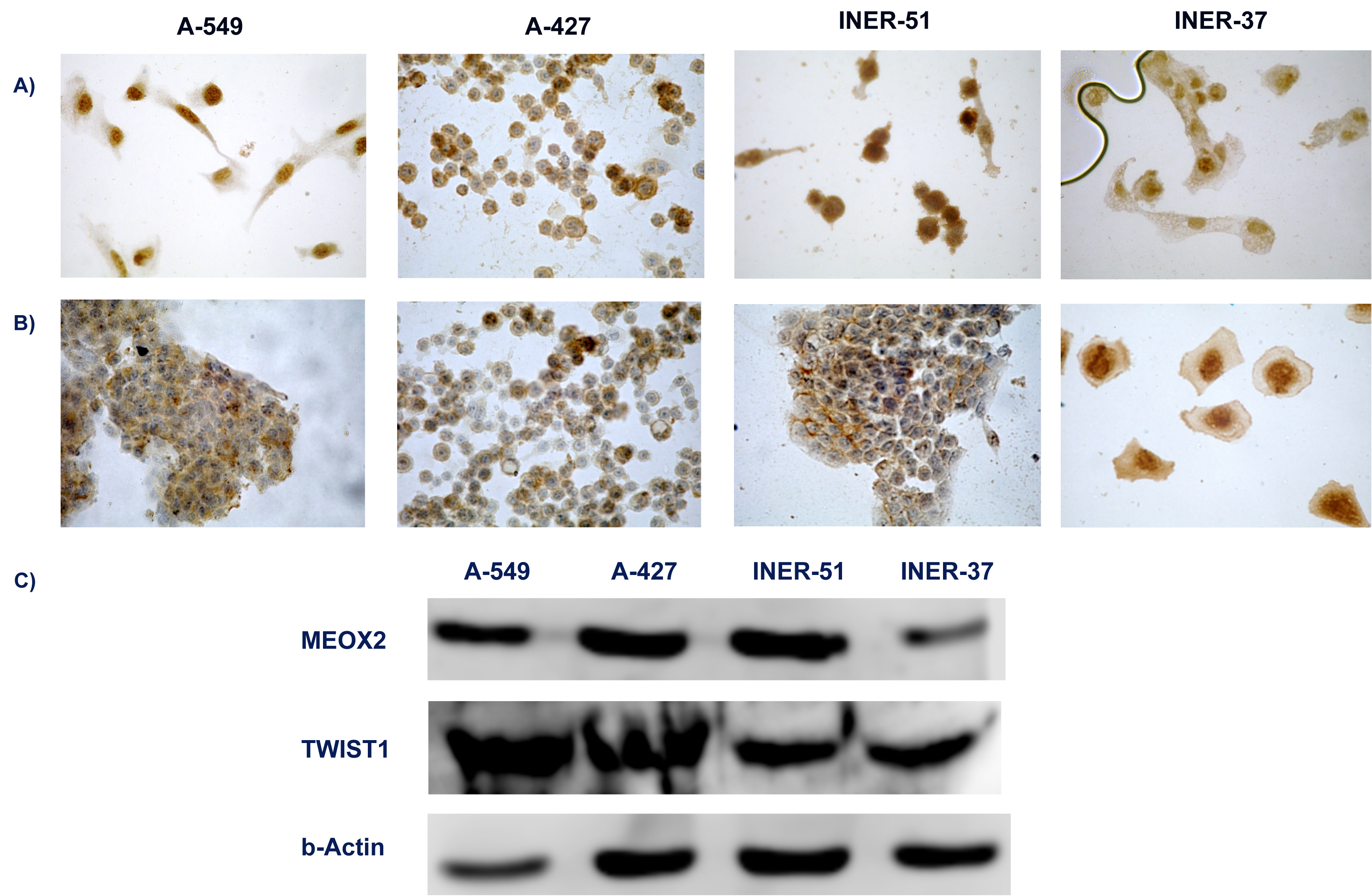

Supplement: Figure S4 — MEOX2 and TWIST1 protein expression analysis in Non-Small Cell Lung Carcinoma cell lines. (A) Immuno-histochemical Protein MEOX2 expression, (B) Immuno-histochemical Protein TWIST1 expression (Microphotographs at 200X and 400X), and (C) Western Blot Protein MEOX2 and TWIST1 expression in NSCLC cell lines (A-549, A-427) from ATCC; and (INER-51, INER-37) established from Mestizo Mexican patients. (TIF) [file pone.0114104.s004.tif]

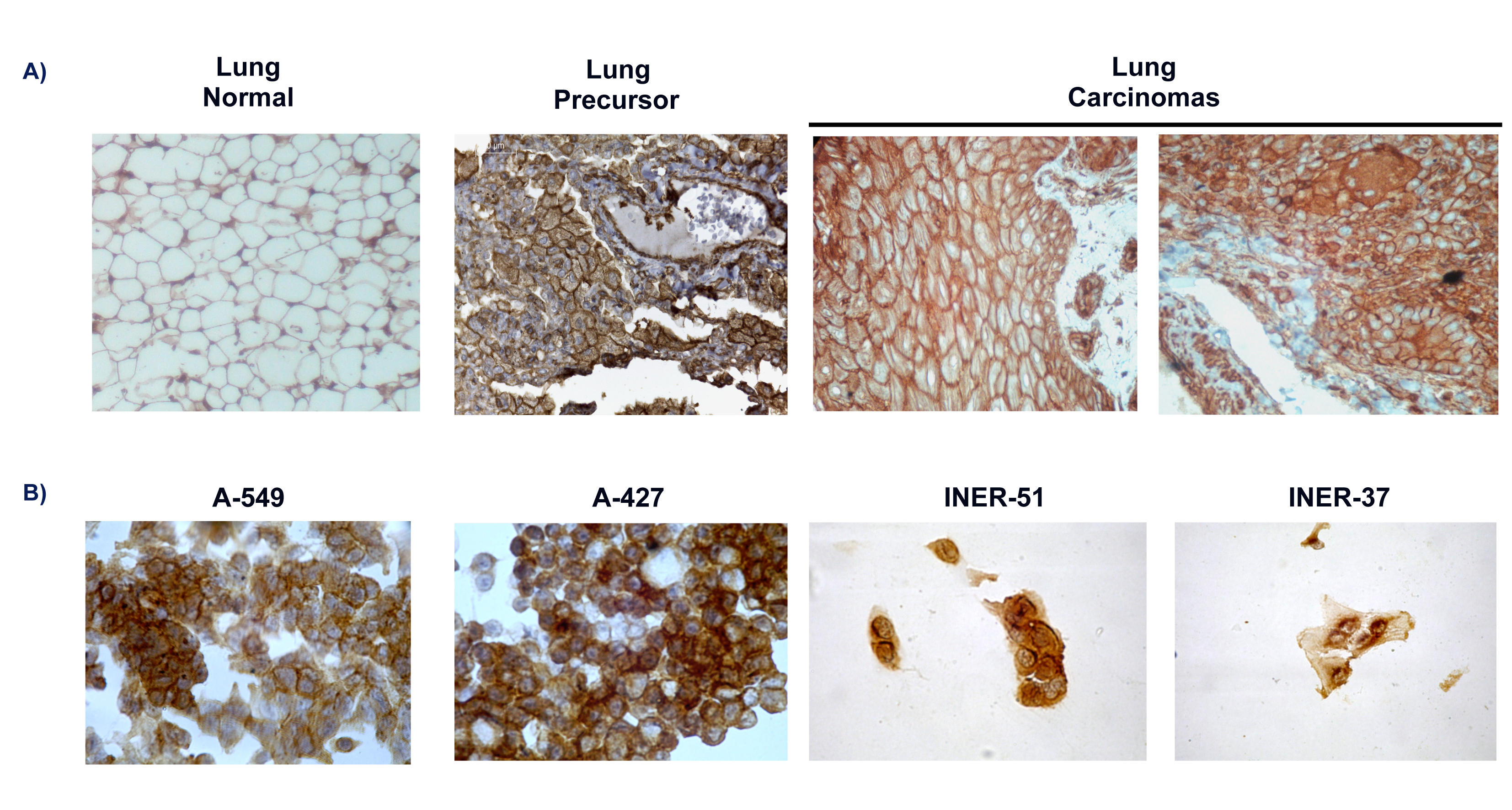

Supplement: Figure S5 — EVX1 protein expression analysis in Lung Normal and Lung Lesion Tissues, as well Non-Small Cell Lung Carcinoma cell lines. (A) EVX1 protein expression in lung normal adjacent, lung precursor and lung carcinoma lesions. Using the Formalin Fixed and Paraffin Embedded (FFPE) method. (B) EVX1 protein expression in NSCLC cell lines A-549, A-427, INER-51, and INER-37 (Microphotographs at 200X and 400X). (TIF) [file pone.0114104.s005.tif]

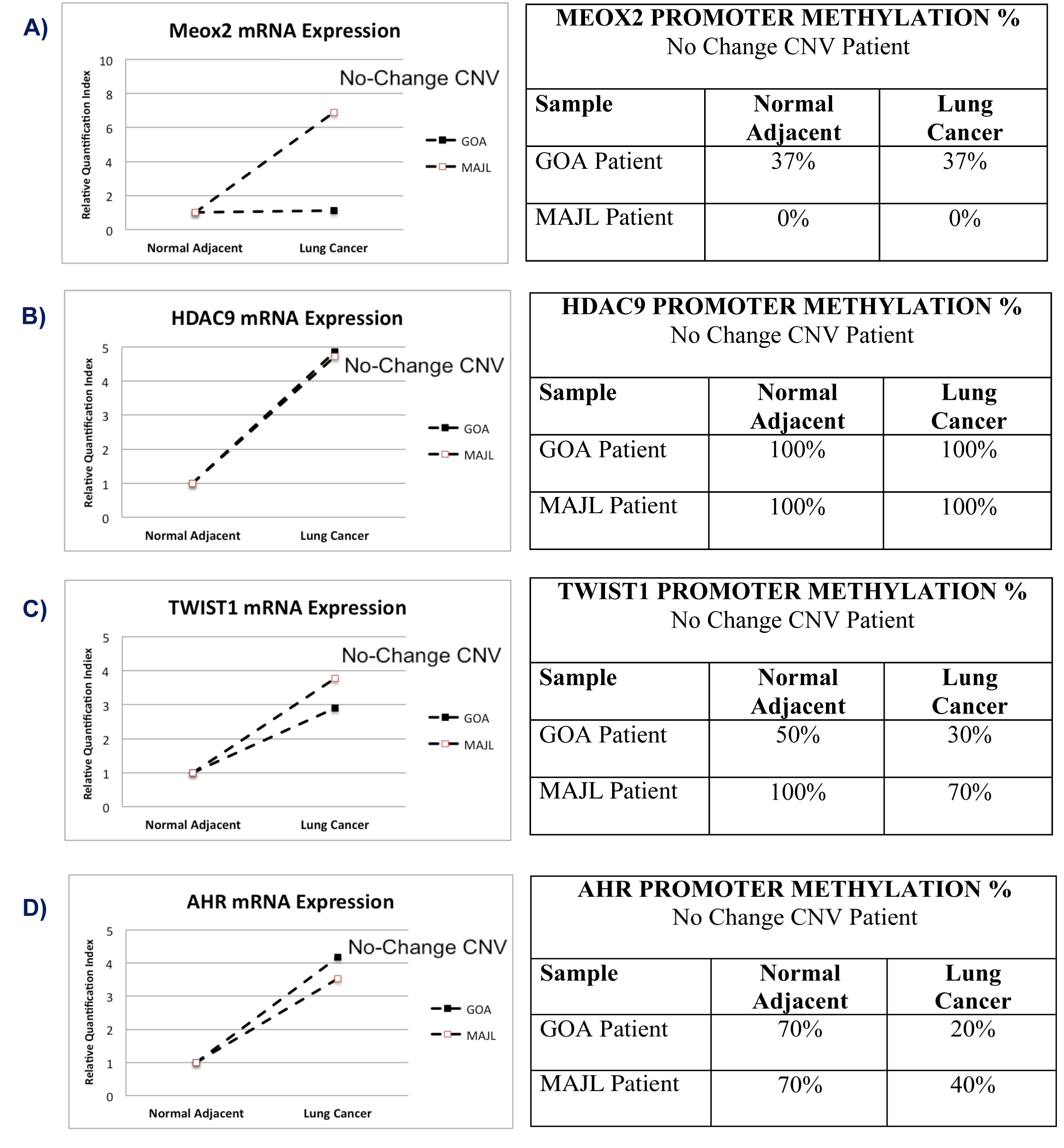

Supplement: Figure S6 — Promoter methylation and mRNA expression paired analysis for no-change CNV patients. Correlation analysis between lung normal adjacent to lung tumor (LNAT), and lung tumor (LT). (A) MEOX2, (B) HDAC9, (C) TWIST1, and (D) AhR for patients without change CNV. (TIF) [file pone.0114104.s006.tif]

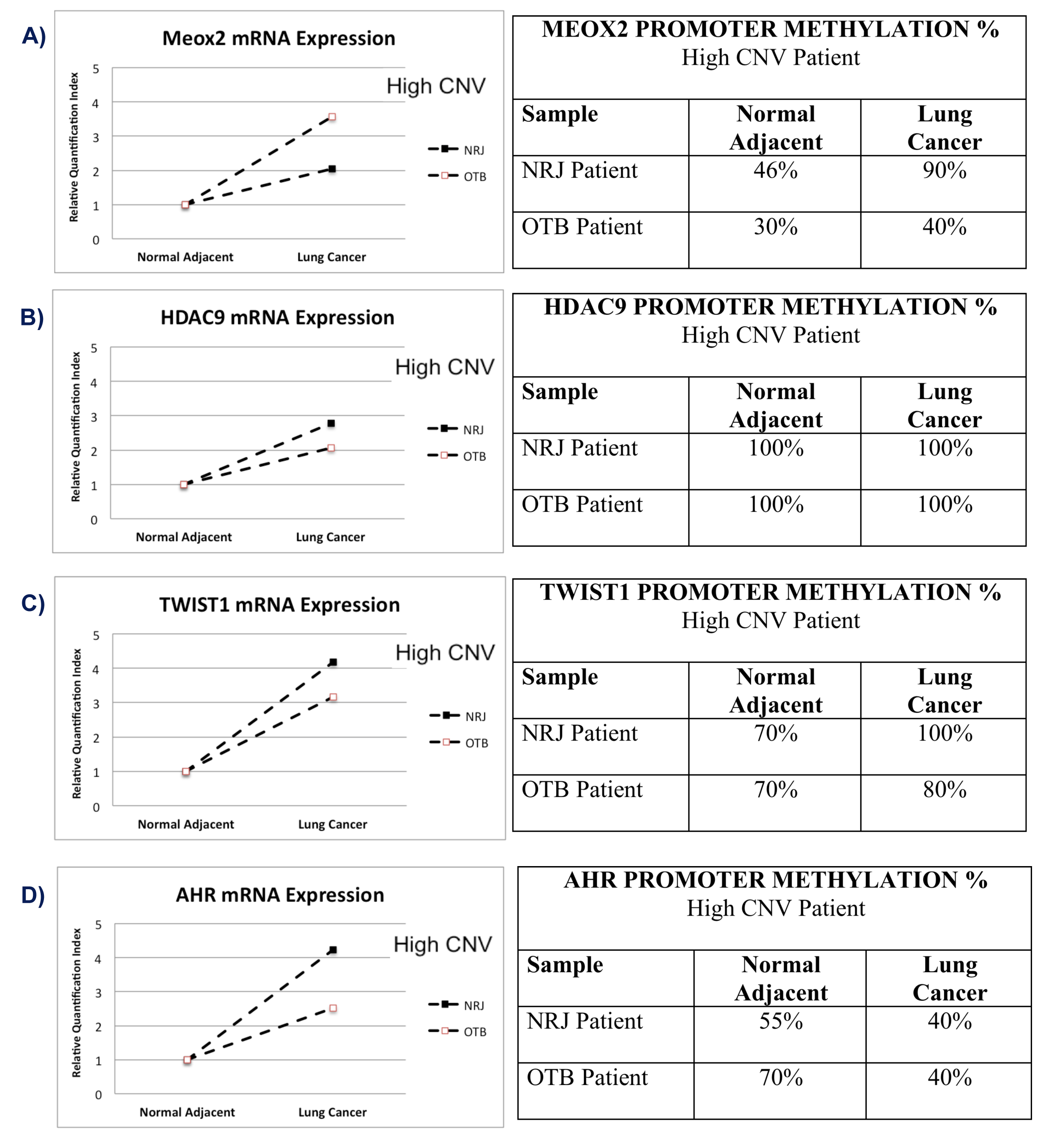

Supplement: Figure S7 — Promoter methylation and mRNA expression paired analysis for high-CNV patients. Correlation analysis between lung normal adjacent to lung tumor (LNAT), and lung tumor (LT). (A) MEOX2, (B) HDAC9, (C) TWIST1, and (D) AhR for patients with high frequency of CNV. (TIF) [file pone.0114104.s007.tif]
