# Supplementary material for: Overexpression of MEOX2 and TWIST1 Is Associated with H3K27me3 Levels and Determines Lung Cancer Chemoresistance and Prognosis
Source: PLoS One. 2014 Dec 2;9(12):e114104. doi: 10.1371/journal.pone.0114104 (PMC4252097; doi:10.1371/journal.pone.0114104)
Supplement: Table S1 — Oligonucleotide sequences for mRNA expression assays. (DOC) [file pone.0114104.s008.doc]

**TABLE S1. Oligonucleotide sequences for mRNA expression assays.**

|  | | | | |
| --- | --- | --- | --- | --- |
| **Gene (mRNA)** | **Sequence 5’ to 3’** | **Region** | **Tm ^o^C** | **Amplicon Size** |
| *MEOX2* | F-TCCTGTGCTCCAACTCTTC  R-CTTCTCAACCTCGTGTCCT | 7p21.2 | 60 | 203pb |
| *HDAC9* | F-CGACAGCAAAAGCTTCTTGTAG  R-GATGTTCTTCGAAAACGACAGC | 7p21.1 | 60 | 192pb |
| *TWIST1* | F-GAGTCCGCAGTCTTACGAG  R-GAGCATTCTGACGCCTGAG | 7p21.1 | 60 | 160pb |
| *AhR* | F-GGCCTGAACTTACAAGAAGG  R-GGAAGAACATTCAAGTCCGG | 7p21.1 | 60 | 215pb |
| *EVX1* | F-CGAGTACCAGCACAGCAAAG  R-GAAACGACACGACCATGAGC | 7p15.2 | 60 | 270pb |
| *β-actin* | F-CGACAGCAAAAGCTTCTTGTAG  R-GATGTTCTTCGAAAACGACAGC | 7p22.1 | 60 | 210pb |
