# Supplementary material for: Overexpression of MEOX2 and TWIST1 Is Associated with H3K27me3 Levels and Determines Lung Cancer Chemoresistance and Prognosis
Source: PLoS One. 2014 Dec 2;9(12):e114104. doi: 10.1371/journal.pone.0114104 (PMC4252097; doi:10.1371/journal.pone.0114104)
Supplement: Table S2 — Oligonucleotide sequences for promoter analysis. (DOC) [file pone.0114104.s009.doc]

**TABLE S2. Oligonucleotide sequences for promoter analysis.**

|  | | | |
| --- | --- | --- | --- |
| **Gene** | **Primer Sequence** | **Tm ^o^C** | **Product size** |
| *F_MEOX2* | 5'-GGAATCAGGGTGCTAGGTTG-3' | 55 | 330bp |
| *R_MEOX2* | 5'-CCCAGAGAACTGCTTTCAGG-3' |
| *F_HDAC9* | 5´-AAGCACGTTCCTATTTCCC-3´ | 55 | 304bp |
| *R_HDAC9* | 5´-TGTCTGCAGCATATTCCAG-3 |
| *F_TWIST1* | 5'-TGAGACATCACCCACTGTGTAG-3' | 55 | 288pb |
| *R_TWIST1* | 5'-TCGGGGTCTAACAATTCGTCCTCCCAA-3' |
| *F_AhR* | 5'-CAGTCCCATTGGTTGTCTAC-3' | 55 | 252pb |
| *R_AhR* | 5´-TTCCATTCCGTCTTCCTTG-3´ |
