# Supplementary material for: Overexpression of MEOX2 and TWIST1 Is Associated with H3K27me3 Levels and Determines Lung Cancer Chemoresistance and Prognosis
Source: PLoS One. 2014 Dec 2;9(12):e114104. doi: 10.1371/journal.pone.0114104 (PMC4252097; doi:10.1371/journal.pone.0114104)
Supplement: Table S3 — Restriction sites and combination of restriction enzymes. (DOC) [file pone.0114104.s010.doc]

**TABLE S3. Restriction sites and combination of restriction enzymes.**

|  |  | **Restriction sites** | | |  |
| --- | --- | --- | --- | --- | --- |
| **Gene** | **Sequence (5'-3')** | ***HpaII***  **(5'-*CCGG*-3')** | ***HpyCH4IV***  **(5'-*ACGT*-3')** | **AciI**  **(5'-CCGC-3')** | |
| *MEOX2* | GGAATCAGGGTGCTAGGTTGGGGGTGGTGTGCG**CCGC**GCCCAGGCCTCCAGAGTCTGGAGGGCAGAGCTGCTGCCCTC***CCGG*GCGG**GACTTAG***ACGT***TGGCGTG**CCGC**TTGTAGTCCTGGCAG***CCGG***CAGTGCTGG**GCGG**GCGTGGACGCTCT***ACGT***CCGTCCAGCAGGAGATAGGAAGTGGAACTGGGAGCGCGTGGCGCAGGCTGA**GCGG**GAGTTTTGGAGGGAGTTTGCATGTGGTCAACTCTGGGCTCCTAAGCCAGCCCCCAGCTC***ACGT***TACACTCTATTTATAACTTCTCAGAGCCATTTCCCCCTGAAAGCAGTTCTCTGGG | 2 NA | 3 | 5 | |
| *HDAC9* | AAGC***ACGT***TCCTATTTCCCACCTGCTTGTAGTTT***CCGG***GATAACCTAAACTCCAGAGAGCTATAGCATCCACTCTGTCCTTTCTGCTTTGCACACAGGTTGGTAACATGGGAAAAGTGTCCAGGTCTTTTTAAAAGTGGATGCCCATTTGAGCAGAAAGGAAATCATTGTCGAAGTTGATCCTCTGCTGCTTCTCCTCAGGGAGGAGGGAGAACCAGCGAGGGTAGCTCCTGGGG***CCGG***TGCACTGAGCAGTGATGAATGTTTCATGTAGCTGAAGTAAGAGTGACTGGAATATGCTGCAGACA | 2 | 1 NA | NA | |
| *TWIST1* | TGAGACATCACCCACTGTGTAGAAGCTGTTGCCATTGCTGCTGTCACAGCCACT***CCGG***ATGGGGCTGCCA**CCGC**GGCCAGGACAGTCTCCTCCGA**CCGC**TTCCTGGGCTGCGCTAGGGTTCGGGGGCGCTGC**CCGC**ACGCT***CCGG***CGGGGAAGGAAATCGCC**CCGC**GC**CCG*CCGG***AGGAAGGCGACGGGGAGGGAAGGGGGAGGGCGGCTAGGAGGCGGGTGGAGGGG***CCGG*CCGC*CCGG***GCCAGGTCGTTTTTGAATGGTTTGGGAGGACGAATTGTTAGACCCCGA | 5 | NA | 6 | |
| *AhR* | CAGTCCCATTGGTTGTCTACCAGTTGTCTACCAGTTAATTGTAATTCTTAGCCACAAGTTAGCTGACCCACCGTCTCTCAAACAGGTGAAGTTAAGCTCAATATTTGGCATATCTTGATATACTTTGTACAGTCTAGCTACAATAAGTTTGCCTATGCACGAAGATGGCTA***CCGG***CGGGGGGGGGCGTCCTT***ACGT***CCT***ACGT***CATC***ACGT***G***CCGG***GATGAGGGTGGGGCCCTCAAGGAAGACGGAATGGAA | 2 | 3 | NA | |

(N.A.): Not analyzed.
