# Supplementary material for: Overexpression of MEOX2 and TWIST1 Is Associated with H3K27me3 Levels and Determines Lung Cancer Chemoresistance and Prognosis
Source: PLoS One. 2014 Dec 2;9(12):e114104. doi: 10.1371/journal.pone.0114104 (PMC4252097; doi:10.1371/journal.pone.0114104)
Supplement: Table S4 — High frequency micro-aberrations detected at 7p in lung cancer patients. (DOC) [file pone.0114104.s011.doc]

**TABLE S4. High frequency micro-aberrations detected at 7p in NSCLC patients.**

| **Chr Ubication** | **Start** | **End** | **Length (bp)** | **RefSeq ID** | **Gene Symbol** | **Lung Cancer Frequency %** |
| --- | --- | --- | --- | --- | --- | --- |
| *7p22.3* | 1573494 | 1576155 | 18891 | NM_032302 | *PSMG3* | *68* |
| *7p22.3* | 1620631 | 1622854 | 117089 | NR_001288 | *TFAMP1* | *72* |
| *7p22.3* | 1750632 | 1754116 | 99786 | NM_001128636 | *ELFN1* | *72* |
| *7p22.1* | 6694588 | 6713091 | 26920 | NM_006956 | *ZNF12* | *56* |
| *7p21.3* | 7188770 | 7250506 | 856449 | NM_020156 | *C1GALT1* | *60* |
| *7p21.3* | 7643099 | 7724763 | 856449 | NM_002947 | *RPA3* | *60* |
| *7p21.3* | 7974947 | 8095234 | 45496 | NM_138426 | *GLCCI1* | *60* |
| *7p21.2* | 13897380 | 13995289 | 3348707 | NM_004956 | *ETV1* | *60* |
| *7p21.2* | 14151198 | 14847600 | 3348707 | NM_004080 | *DGKB* | *60* |
| **7p21.2** | **15617361** | **15692833** | **3348707** | **NM_005924** | ***MEOX2*** | **60** |
| *7p21.1* | 16097683 | 16427472 | 3348707 | NM_001101417 | *LOC729920* | *60* |
| *7p21.1* | 16652283 | 16712672 | 150674 | NM_014038 | *BZW2* | *56* |
| *7p21.1* | 16759875 | 16790686 | 38005 | NM_014399 | *TSPAN13* | *60* |
| *7p21.1* | 16797959 | 16811133 | 72616 | NM_006408 | *AGR2* | *60* |
| *7p21.1* | 16865554 | 16888138 | 1213086 | NM_176813 | *AGR3* | *60* |
| **7p21.1** | **17304831** | **17352299** | **1213086** | **NM_001621** | ***AHR*** | **60** |
| *7p21.1* | 18032924 | 18034011 | 1213086 | NM_175886 | *PRPS1L1* | *60* |
| **7p21.1** | **18501893** | **19003517** | **651318** | **NM_178423** | ***HDAC9*** | **60** |
| **7p21.1** | **19121615** | **19123820** | **101430** | **NM_000474** | ***TWIST1*** | **60** |
| *7p21.1* | 19150929 | 19151569 | 101430 | NM_152898 | *FERD3L* | *60* |
| *7p15.3* | 19701609 | 19715185 | 1364459 | NM_001002926 | *TWISTNB* | *60* |
| *7p15.3* | 20653740 | 20761821 | 76919 | NM_178559 | *ABCB5* | *60* |
| *7p15.3* | 20788419 | 20793030 | 113248 | NM_182700 | *SP8* | *60* |
| *7p15.3* | 21434213 | 21520676 | 174422 | NM_003112 | *SP4* | *60* |
| *7p15.3* | 21907041 | 21952042 | 832114 | NM_018719 | *CDCA7L* | *60* |
| *7p15.3* | 22124432 | 22363058 | 832114 | NM_012294 | *RAPGEF5* | *60* |
| *7p15.3* | 22733342 | 22738145 | 329685 | NM_000600 | *IL6* | *56* |
| *7p15.2* | 26196080 | 26206938 | 124838 | NM_002137 | *HNRNPA2B1* | *56* |
| *7p15.2* | 26207848 | 26219501 | 124838 | NM_016587 | *CBX3* | *56* |
| *7p15.2* | 27099138 | 27102150 | 247715 | NM_153620 | *HOXA1* | *56* |
| *7p15.2* | 27106497 | 27108919 | 247715 | NM_006735 | *HOXA2* | *56* |
| *7p15.2* | 27112333 | 27120141 | 247715 | NM_153632 | *HOXA3* | *56* |
| *7p15.2* | 27134650 | 27136924 | 247715 | NM_002141 | *HOXA4* | *56* |
| *7p15.2* | 27147520 | 27149812 | 247715 | NM_019102 | *HOXA5* | *56* |
| *7p15.2* | 27159862 | 27162821 | 247715 | NM_006896 | *HOXA7* | *56* |
| *7p15.2* | 27168581 | 27171674 | 247715 | NM_152739 | *HOXA9* | *56* |
| *7p15.2* | 27176734 | 27180480 | 247715 | NM_018951 | *HOXA10* | *56* |
| 7p15.2 | 27203023 | 27206250 | 247715 | NM_000522 | *HOXA13* | *56* |
| **7p15.2** | **27248688** | **27252717** | **247715** | **NM_001989** | ***EVX1*** | **56** |

Bold case indicates genes selected for the validation study.
